# Effect of plant waste materials as pore-forming agents on the preparation and characterization of macroporous cordierite–mullite–zirconia ceramic composites

**DOI:** 10.1186/s13065-025-01696-8

**Published:** 2025-12-19

**Authors:** Nadeen Nasser, Mohamed M. S. Wahsh, M. S. Rizk, Gehad G. Mohamed, Omar A. Fouad

**Affiliations:** 1 Chemistry Department, Faculty of Science, Cairo University, Giza, 12613 Egypt; 2 Nanoscience Department, Faculty of Basic and Applied Science, Egypt-Japan University of Science and Technology, New Borg El Arab, 21934 Alexandria Egypt; 3 Refractories, Ceramics and Building Materials Department, National Research Centre, Cairo, 12622 Egypt

**Keywords:** Nanostructure, Cordierite, Zirconia, Mullite, Porous ceramics, Microstructure

## Abstract

Porous ceramic materials have generated significant attention and played pioneering roles in a variety of industries. This work aims to prepare porous ceramic composites based on cordierite, mullite, and tetragonal zirconia (t-ZrO_2_), which are considered one of the most effective materials in several applications. Nanoparticles were synthesized using sol-gel and co-precipitation methods and then characterized using X-ray diffraction (XRD), transmission electron microscope (TEM), and surface area measurements. The tetragonal form of the prepared nano zirconia was stabilized using ceria. To prepare the cordierite–mullite–t-ZrO_2_ macroporous ceramics, nano t-ZrO_2_ was added in various ratios from 0 to 30 wt% at the expense of nano mullite, while nano cordierite was added at a 70 wt%. Plant waste materials (bagasse ash and sawdust ash) were recycled and used as pore-forming agents in the fabrication of porous ceramic materials at a constant amount of 10 wt% to increase porosity and reduce environmental pollution problems. The prepared samples were sintered at three different temperatures: 1375, 1400, and 1350 °C. The densification characteristics (bulk density and apparent porosity), cold crushing strength (CCS), phase composition, microstructure, pore size distribution, and contact angle of sintered porous ceramics were studied. The results showed that the bulk density, apparent porosity, and cold crushing strength ranged from 1.42 to 1.95 g/cm^3^, 46 to 56%, and 3.26 to 31.35 MPa, respectively.

**Supplementary Information:**

The online version contains supplementary material available at 10.1186/s13065-025-01696-8.

## Introduction

It is widely acknowledged that the preparation of composite substances (ceramic composites), metal-organic frameworks (MOFs), covalent organic frameworks (COFs), and conjugated polymers is an effective method for synthesizing materials with characteristics unattainable by monolithic materials due to their exceptional properties [1, 2].

One of the most essential composite materials is ceramic composite materials which are ideal for industrial and high-temperature implementations [3, 4]. Ceramic materials have grown in popularity and countless attention nowadays because of their qualities that fit different applications and one of the most essential scopes is porous ceramic materials [5]. Porous ceramic materials are mainly characterized by high pore density after firing. They are also used for various purposes due to their unique qualities, like their substantial chemical and thermal resilience, corrosion resistance, low-weight structural components, and low thermal conductivity [6]. They are applied in different applications as filters, membranes [7], catalysis [8], insulation, and hot corrosive gas filtration [4].

Nanoceramic materials are also attractive for usage in a variety of applications because of their strength, hydrophilicity, biocompatibility, insulating and/or conducting qualities, and resistance to wear. The utilization of nanocrystalline ceramic particles in the production of uniform and irregular green compacts leads to the formation of tiny pores at the nanometer scale that possess great mobility. As a result, the process of densification occurs more rapidly. The reduced nanoparticle size enhanced particle interaction and enabled matter diffusion along grain boundaries, promoting grain development in crystals during the solid-state sintering process and ultimately improving the sintering process [9]. Furthermore, there is a direct correlation between the average pore size of a material and its average particle size. The primary benefit of utilizing nanocrystalline ceramic powders for manufacturing ceramic materials is their ability to create both dense and porous structures [9, 10]. The strategic importance of using nanoparticles lies in constructing macroporous ceramic composites, because these nanoparticles successfully decouple the typical porosity-strength trade-off by leveraging the enhanced sinterability of nanoscale powders to form dense, strong pore walls within a highly porous macro-structure. The resulting composites exhibit a synergistic combination of high apparent porosity, satisfactory mechanical strength, and functional hydrophilicity [11, 12].

Cordierite (Mg_2_Al_4_Si_5_O_18_) is considered one of the ceramic materials that can qualify for these outstanding properties and to be a pioneer in diverse fields due to its high mechanical properties, good stability toward thermal and chemical circumstances, high refractoriness, high stability toward corrosive conditions, low dielectric constant, and modest thermal expansion, between (1–2)×10^− 6^ °C^− 1^ [13, 14]. So, it is broadly used in industries, electronic engineering, insulation, and catalyst support [15]. Cordierite ceramics have a MgO: Al_2_O_3_:SiO_2_ system from ternary components in a 2:2:5 ratio [13, 16]. Inappropriately, the most common drawbacks of cordierite are its relatively low thermal stability and mechanical properties, which limit its applications. Consequently, incorporating an additional phase that combines with the boundaries of the cordierite can significantly improve a material’s mechanical and thermal properties. Consequently, forming composites is a decent solution to overcome the drawbacks of a single phase and enhance the required properties designed for fitting industrial applications. Mullite and t-ZrO_2_ are the best choices for enhancing cordierite’s thermal stability and mechanical strength [17, 18].

In the aluminosilicate system, mullite is the stable crystalline phase at temperatures between room temperature to high temperatures at ambient atmospheric pressure, and the stoichiometric composition of mullite is 2Al_2_O_3_–SiO_2_ or 3Al_2_O_3_–2SiO_2_ [19, 20]. Porous mullite ceramics have attracted a lot of focus because of their remarkable features, including high melting points, outstanding resistance to heat shock, and excellent chemical stability. These features make porous mullite attractive as lightweight structural materials, metal molten ash filters, and catalyst carriers [20, 21]. Consequently, ceramic-based cordierite and mullite can resist severe temperatures and insulation. Therefore, they are widely used in different applications relating to refractories and furnaces [22, 23].

The nano tetragonal-zirconia ceramic (TZC) material is used owing to its remarkable contribution to developing fracture toughness, corrosion resistivity, and chemical constancy [24, 25]. There are three phases of zirconia: cubic, tetragonal, and monoclinic [4, 26]. The temperature range and the stabilizer amount influence the synthesis of the specific phase. The stability of the monoclinic phase is from room temperature to 1173 °C. The tetragonal phase is prepared at temperatures from 1173 to 2370 °C, whereas around 2370 °C is the stable temperature range for the cubic phase [9, 25]. From a practical standpoint, tetragonal-zirconia ceramic (TZC) is the best material to use in various applications. Consequently, zirconia stabilization at the tetragonal phase at room temperature without phase transition to monoclinic phase, oxide stabilizers should be used such as ( Calcia, Magnesia, Yttria, Ceria,…) [27]. Ceria (CeO_2_) is used as a dopant to help the tetragonal zirconia phase maintain its required stability at room temperature rather than changing into the monoclinic phase [24, 28]. Hence, It has been demonstrated that the incorporation of distinct grains-based metastable tetragonal-ZrO_2_ enhances the ceramic matrix’s mechanical characteristics, particularly its ability to withstand fracture, which expands its range of applications as a primary material [29, 30].

Accordingly, adding nano mullite and nano t-ZrO_2_ into nano cordierite significantly enhances cordierite porous ceramic materials’ properties. Additionally, the pore-forming agents’ addition will also affect the general characteristics of porous composites. Also, one of the common ways to create porous ceramics with regulated microstructure (porosity and pore size) involves employing pore-forming substances. These pore-forming substances burn off when ceramics are heated to their last firing temperatures, causing empty holes [43]. Nowadays, waste materials such as sugarcane bagasse and wood sawdust represent a disastrous problem because of their enormous worldwide output. These unused materials are considered the best and cheapest pore-forming agents. Consequently, they are recycled to overcome their impacts [13, 31]. Thus, after studying the individual properties of cordierite, mullite, and zirconia, this work presents a novel approach through the strategic design and synthesis of a ternary macroporous composite that utilizes the advantages of each phase at the nanoscale combined with two distinct plant waste ashes (bagasse and sawdust) as sustainable pore-forming agents and studying the densification parameters, linear change, microstructure, pore size distribution, cold crushing strength, and phase composition of the sintered samples. This study investigated (1) the enhancement of a cordierite matrix through the incorporation of nano-mullite and nano-t-ZrO₂ such as transformation toughening (2) the use of individually synthesized nanoparticles to improve sinterability at lower temperatures compared to conventional solid-state methods; (3) the systematic replacement of mullite with zirconia (from 0 to 30 wt%) to determine optimal compositions at optimum sintering temperature that balance porosity and mechanical strength; and (4) a comparative analysis of two distinct plant waste ashes (bagasse and sawdust) as sustainable pore-forming agents, linking their chemical composition to the final microstructure and properties, including hydrophilicity for potential filtration applications.

## Materials and experimental data

### Materials and instruments

All the chemicals, reagents, and equipment used are covered in supplemental data. Also, the chemical composition (wt% %) of pore-forming agents (bagasse ash, which is unprocessed sugarcane bagasse acquired from a boiler in an Egyptian sugar refinery in Minya, and sawdust ash, which is raw sawdust ash gained from Furniture Factory in Egypt) was supplied in Supplementary Table 1.

### Methods of preparation

#### Synthesis of nano cordierite (2MgO: 2Al_2_O_3_: 5SiO_2_)

The sol-gel technique was utilized to prepare cordierite nanoparticles. A 1:4 volume ratio of distilled water and ethanol was combined with TEOS and then stirred at 80 °C at 40 rpm till complete hydrolysis of silica. After that, the hexahydrate solutions of aluminum chloride (AlCl_3_·6H_2_O) and magnesium chloride (MgCl_2_·6H_2_O) were added in the stoichiometric proportion of cordierite phase to the hydrolyzed TEOS; hence, the prepared solution was stirred for about one hour. After mixing, an ammonia solution was added drop by drop, and the mixed solution’s pH was maintained at 10 [32]. The precipitates were collected by filtering the solution through ashless filter paper (Whatman^®^ Grade 41, England). The precipitates were then repeatedly washed with distilled water to get rid of the chloride ions. After being dried for 24 h at 150 °C, the precipitates were calcined for 60 min at temperatures of 800, 1000, 1200, and 1350 °C at a 5 °C/min heating rate [33]. The resulting nanopowders were analyzed using TEM and BET surface area techniques to determine the particle size and surface area. XRD was used to determine the calcined temperature that produced pure cordierite.

#### Synthesis of nano mullite (3Al_2_O_3_·2SiO_2_)

For preparing nano mullite by sol-gel method, ethanol and distilled water were mixed with TEOS solution in a 1:4 volume ratio. Then, the prepared solution was stirred at constant stirring at 80 °C till complete hydrolysis of SiO_2_. After hydrolysis, 1 M solution of aluminum chloride hexahydrate (AlCl_3_·6H_2_O) was added to the previous solution in the stoichiometric proportion of the mullite phase and left to mix for one hour by continuous stirring. To precipitate the solution, ammonia solution was added drop by drop till pH 10. The precipitates were obtained by filtering the solution through Whatman^®^ Grade 41, England, ashless filter paper. Chloride ions were extracted from the mixture by washing it three times with distilled water. After being dried for 24 h at 150 °C, ground particles were calcined for 60 min at 1000 °C. The composition of the prepared nanoparticles was determined by XRD analysis, and TEM determined the average particle size and surface area and BET analysis [34].

#### Synthesis of nano tetragonal zirconia (t-ZrO_2_)

Co-precipitation was employed to synthesize nanoparticles of ceria-stabilized tetragonal zirconia (Ce-TZP). Zirconyl chloride octahydrate (ZrOCl_2_·8H_2_O) was dissolved in distilled water to prepare a 1 M solution, and then cerium nitrate hexahydrate solution was added in various weight percentages of 12, 14, and 15%. The previous solutions were named T1, T2, and T3, respectively. Constant stirring was maintained to get homogenous solutions for 60 min. Ammonium chloride solution was added dropwise to the prepared solution to get the precipitate. Adding ammonia solution was discontinued at pH 10.5. The filter paper (Whatman^®^ Grade 41, England) was used to get the precipitate, then washed with distilled water several times. After that, precipitates were dried at 150 °C in the drier for 24 h and then calcined at different temperatures for 60 min. The phase composition of the produced nanoparticles was determined by XRD analysis, and the average particle size and surface area were evaluated by TEM and BET analyses [35].

#### Preparation of pore-forming agent

##### Preparation of bagasse ash

Bagasse was fired at 700 °C for an hour without air, at a rate of 10 °C per minute of heating, in order to prepare it for use in porous ceramic composites. The calcined bagasse was ground and run through 90 μm sieves after cooling.

##### Preparation of sawdust ash

Sawdust was fired at 500 °C for 60 min at a rate of 10 °C per minute of heating in the absence of air to prepare it for utilization in porous ceramic products. Then, calcined sawdust was crushed and passed through sieves (size 90 μm) after cooling.

#### Preparation of cordierite–mullite–zirconia porous ceramic materials

Table 1 indicates the variation of weight percentages of nano mullite and nano t-ZrO_2_, which were mixed with nano cordierite (70 wt%), and either bagasse ash or sawdust ash (as a pore-forming agent) was added in an additional amount of 10 wt%. The different compositional samples of nano cordierite, nano mullite, and nano t-ZrO_2_ are 70:30:0, 70:20:10, 70:10:20 and 70:0:30, respectively. The samples that used bagasse ash as a pore-forming agent were named.

(Z0-B), (Z1-B), (Z2-B) and (Z3-B), while the other samples that used sawdust ash were designated as (Z0-S), (Z1-S), (Z2-S), and (Z3-S). After preparing the previous compositions, about 10 wt% of water was added to samples for homogenous mixing, and they were pressed at 79 MPa using a uniaxial piston press machine to produce cylindrical samples with a 25.4 mm diameter. After drying the samples at 90 °C for 24 h, they were sintered in an electrical furnace with a heating rate of 5 °C per minute for 60 min at several temperatures: 1350, 1375, and 1400 °C.

**Table 1:** The weight percentages (%) of substances that compose the porous ceramic materials and the extra addition of bagasse ash and sawdust ash

Sample	Nano cordierite	Nano mullite	Nano t-zirconia	Bagasse ash	Sawdust ash		Al_2_O_3_	SiO_2_	CaO
Z0-B	70	30	0	10	–	Weight percentages of materials composing the porous ceramic materials	46.46	47.18	0.25
Z1-B	70	20	10	10	–		39.28	44.36	0.25
Z2-B	70	10	20	10	–		32.1	41.54	0.25
Z3-B	70	0	30	10	–		24.92	38.72	0.25
Z0-S	70	30	0	–	10		45.94	44.44	0.07
Z1-S	70	20	10	–	10		38.76	41.6	0.07
Z2-S	70	10	20	–	10		31.58	38.8	0.07
Z3-S	70	0	30	–	10		24.4	35.95	0.07

P_2_O_5_	SO_3_^−^	Na_2_O	Fe_2_O_3_	K_2_O	MgO	C	ZrO_2_	Other elements	Total
0.18	0.002	0.05	0.44	0.02	9.79	5.62	0	0	110
0.18	0.002	0.05	0.44	0.02	9.79	5.62	10	0	110
0.18	0.002	0.05	0.44	0.02	9.79	5.62	20	0	110
0.18	0.002	0.05	0.44	0.02	9.79	5.62	30	0	110
0.004	0.005	0	0.014	0.009	9.79	9.75	0	0.14	110
0.004	0.005	0	0.014	0.009	9.79	9.75	10.0058	0.114	110
0.004	0.005	0	0.014	0.009	9.79	9.75	20.0058	0.114	110
0.004	0.005	0	0.014	0.009	9.79	9.75	30.0058	0.114	110

## Results and discussion

### Characterization of the synthesized nano cordierite (Mg_2_Al_4_Si_5_O_18_)

#### XRD of the synthesized nano cordierite

**Fig. 1:**
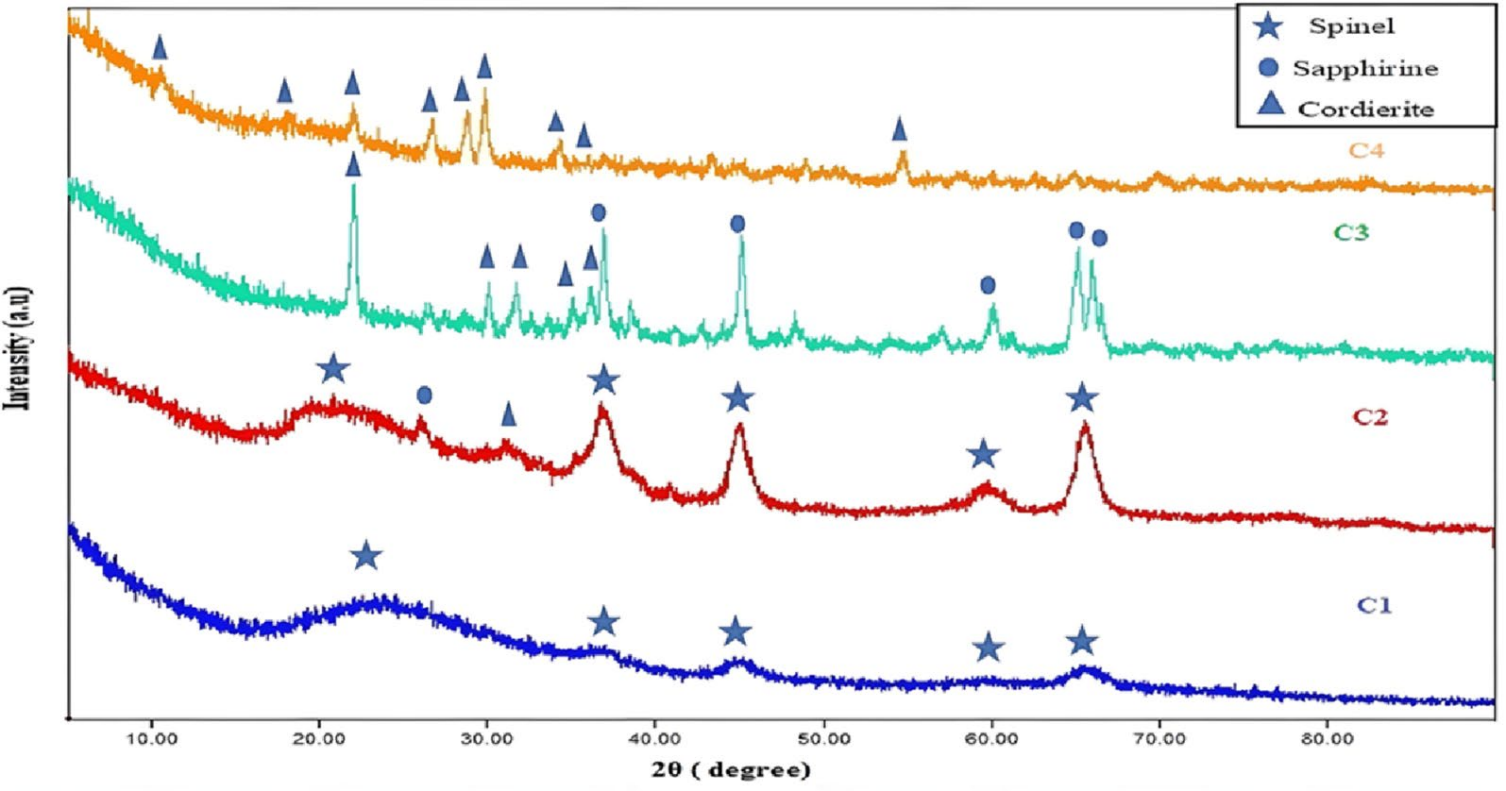
XRD patterns of prepared nano cordierite at various temperatures C1: 800 °C, C2: 1000 °C, C3: 1200 °C and C4: 1350 ° C

Figure 1 indicated the XRD patterns of the prepared nano cordierite at several temperatures of 800, 1000, 1200 and 1350 °C. Pure cordierite was formed at a temperature of 1350 °C according to the XRD pattern. According to JCPDS / PDF Card No.: 13-0294, the orthorhombic structure was concluded, and Cccm (66) was the space group of cordierite nanopowder. The 2θ angles of the peaks of nano-cordierite of 10.5 °, 18.00 °, 19.26 °, 21.95 °, 26.70 °, 28.6 °, 29.87 °, 34.23 °, 39.10 °,43.24 °, and 54.70 ° which correlated to (110), (310), (002), (112), (002), (222), (131), (422), (004), (314), and (624) planes, respectively [36, 37]. The Scherrer equation indicated that the synthesized cordierite’s average crystallite size was 16.89 nm [35].

#### Scanning electron microscopy (SEM) of cordierite nanoparticles

**Fig. 2:**
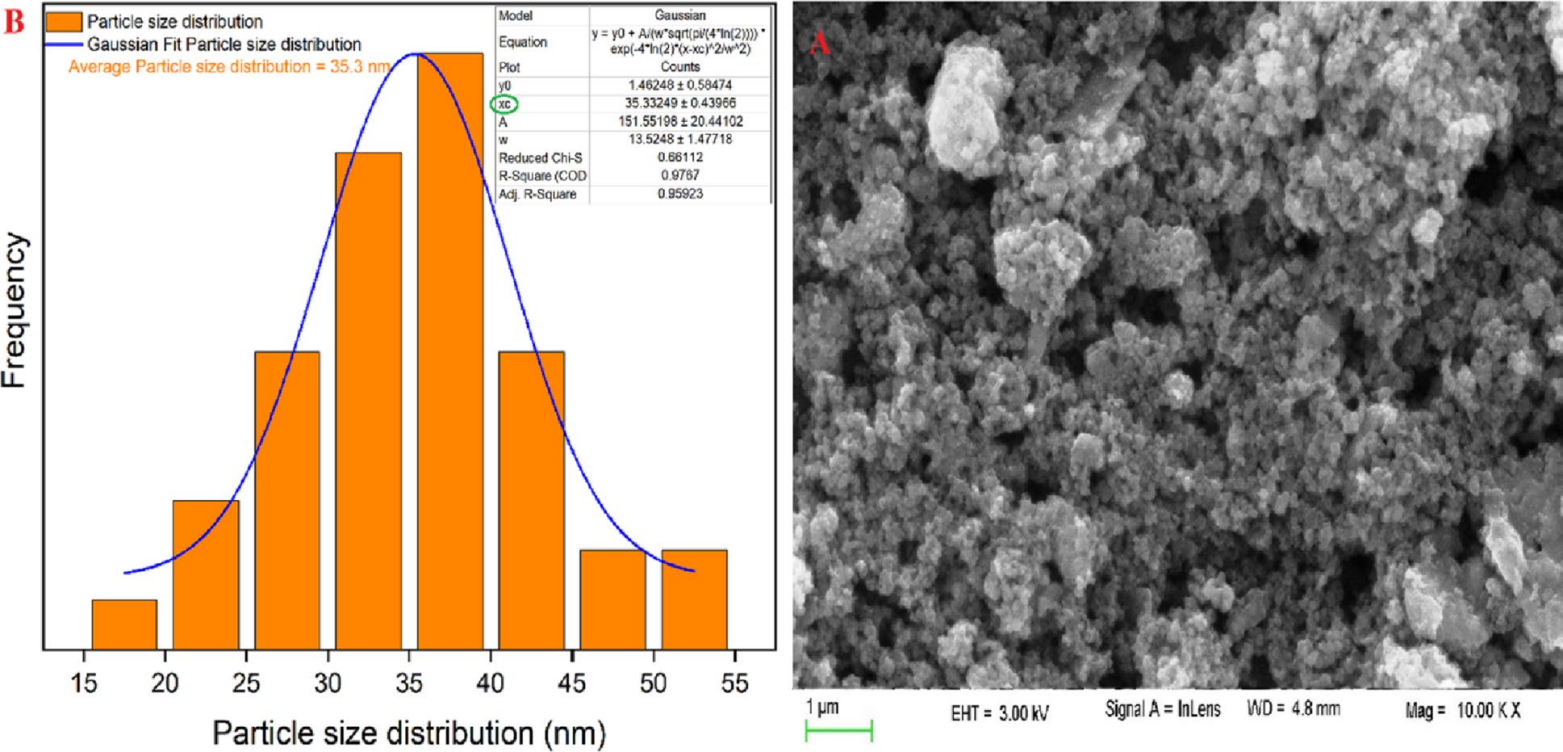
SEM (**A**), particle distribution (**B**) of the nano-synthesized cordierite

Figure 2A presents the SEM pictures of nano cordierite. The synthesized cordierite exhibits a uniform design, spherical morphology, smooth texture, and porous structure. Furthermore, the Gaussian mixture model illustrated the particle size distribution and histogram in Fig. 2B, utilizing ImageJ (1.53e). The produced nano cordierite exhibited an average particle size of 35.3 nm.

#### Transmission electron microscopy (TEM) of nano cordierite

**Fig. 3:**
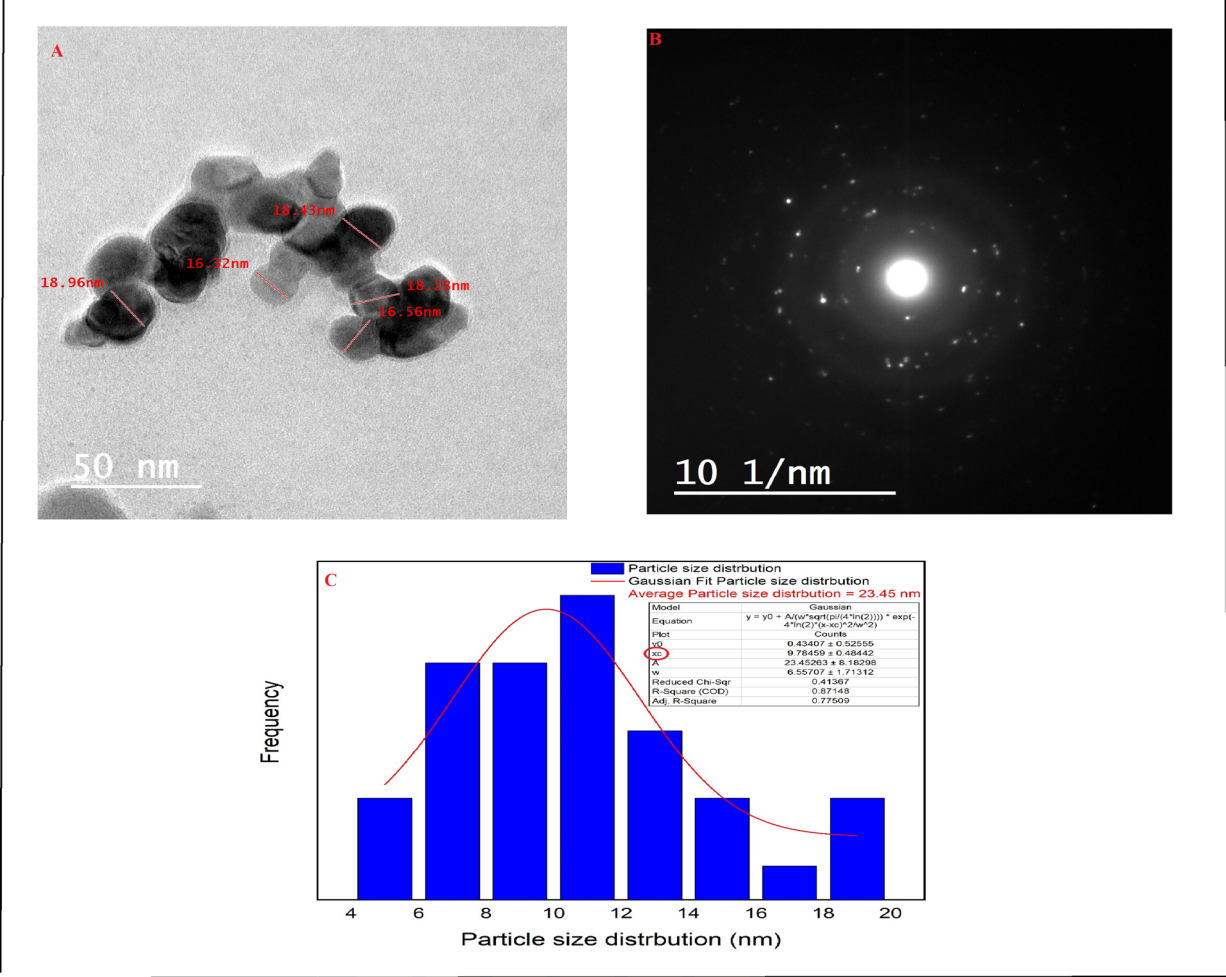
The HR-TEM (**A**), SAED (**B**), and particle size distribution (**C**) of the synthesized cordierite nanoparticles

As shown in Fig. 3A, the TEM image revealed that cordierite particles ranged in size from 16.32 to 18.96 nm, which was acknowledged by the XRD data, which displayed a 16.89 nm average crystallite size. Additionally, the exceptional crystallinity of the synthesized nano cordierite was demonstrated by the precise, practical lattice edge configurations seen in the high-resolution transmission electron microscope (TEM) image of cordierite nanoparticles, as well as in selected area electron diffraction (SAED), as indicated in Fig. 3B [4]. Additionally, the particle size distribution was presented by the Gaussian mixture model and histogram in Fig. 3C using ImageJ (1.53e). It was found that the average particle size is 9.7 nm [33].

#### Brunauer–Emmett–Teller (BET) analysis

**Fig. 4:**
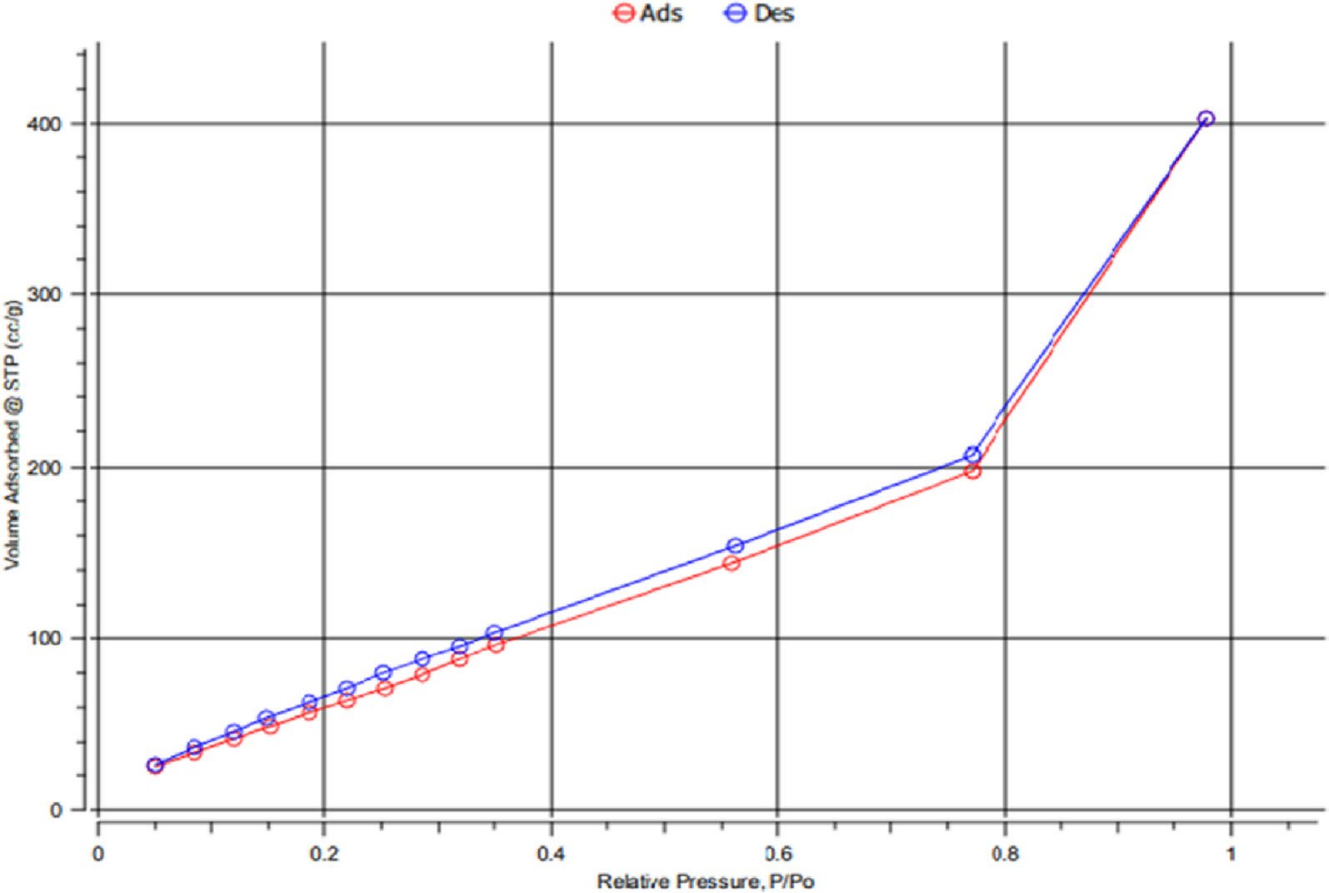
N_2_ adsorption–desorption isotherms for nano cordierite

Surface area, pore volume, and typical pore size of the synthesized nano cordierite were all determined depending on the nitrogen adsorption-desorption isotherm. A type IV-(a)-H3 hysteresis loop isotherm of nano cordierite was demonstrated in Fig. 4 [38, 39]. Additionally, a remarkable improvement in the adsorbed volume at relative pressure P/P_0_ = 0.77 was noticed. The previous data indicate that the total pore volume of nano cordierite was 0.63 cm^3^/g, and its surface area was 332.34 m^2^/g, signifying a high surface area. Furthermore, the average size of the pores was 3.75 nm, representing the mesoporous structure of cordierite nanoparticles [40].

### Characterization of the prepared nano mullite (3Al_2_O_3_·2SiO_2_)

#### XRD of the synthesized nano mullite

**Fig. 5:**
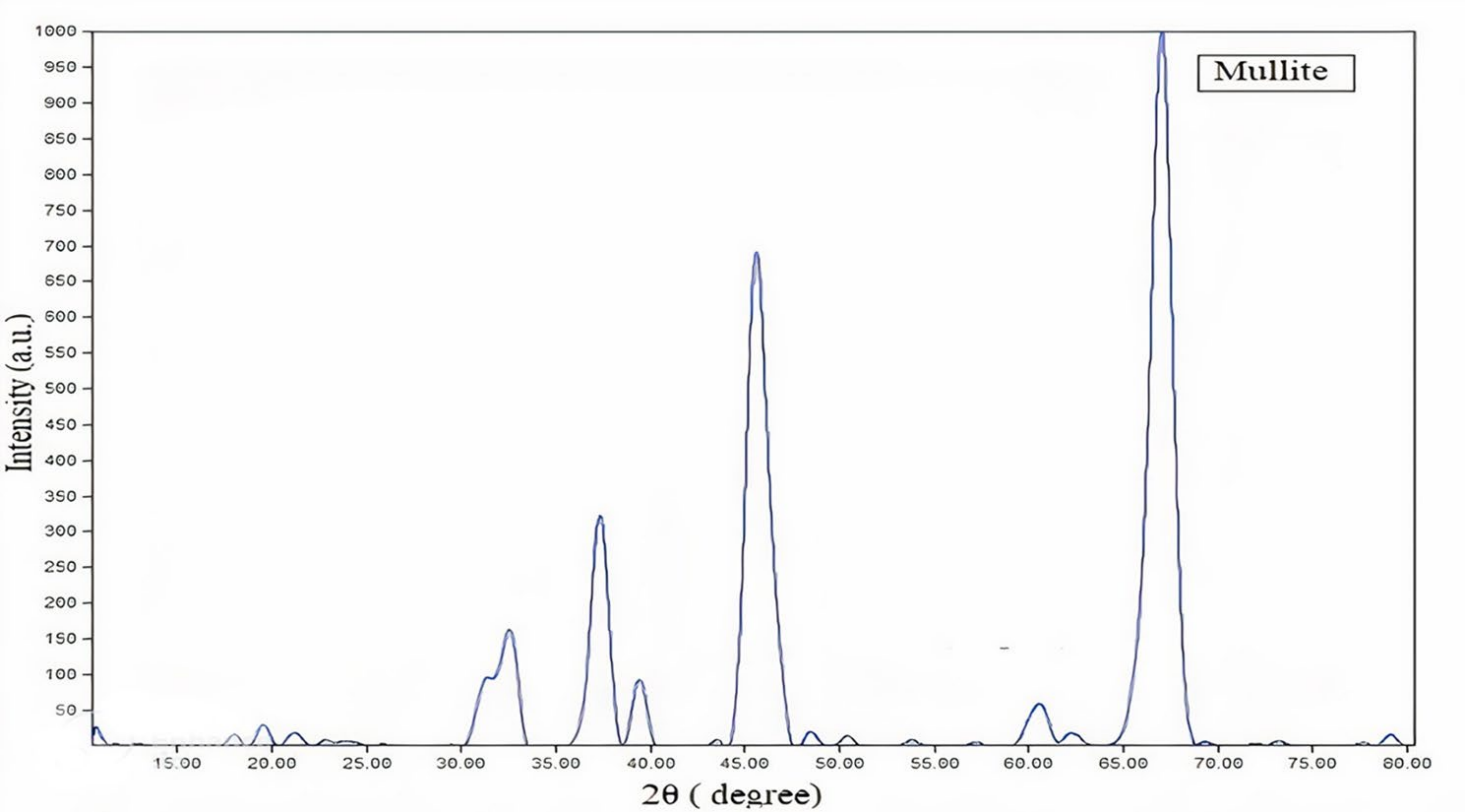
presents the XRD chart of the prepared nano mullite

As exposed in Fig. 5, the pattern of XRD of nano mullite. The XRD pattern confirmed the preparation of mullite through sol-gel method and the mullite’s diffraction peak angles 2θ of 31.06°, 33.07°, 36.84°, 39.04°, 46.23°, 60.24°, and 66.2° were agreed to the (001), (220), (130), (021), (221), (331), and (520) planes, respectively. Additionally, the crystal structure of the nano mullite was orthorhombic, with the space group being Pbam (No. 55) according to the card number.: **JCPDS 15-0776** [34, 41]. Based on the Scherrer equation, the crystallite size of mullite particles could be estimated to be in the range of 5.9 to 8.95 nm, and the average crystallite size was 7.46 nm.

#### Scanning electron microscopy (SEM) of mullite nanoparticles

**Fig. 6:**
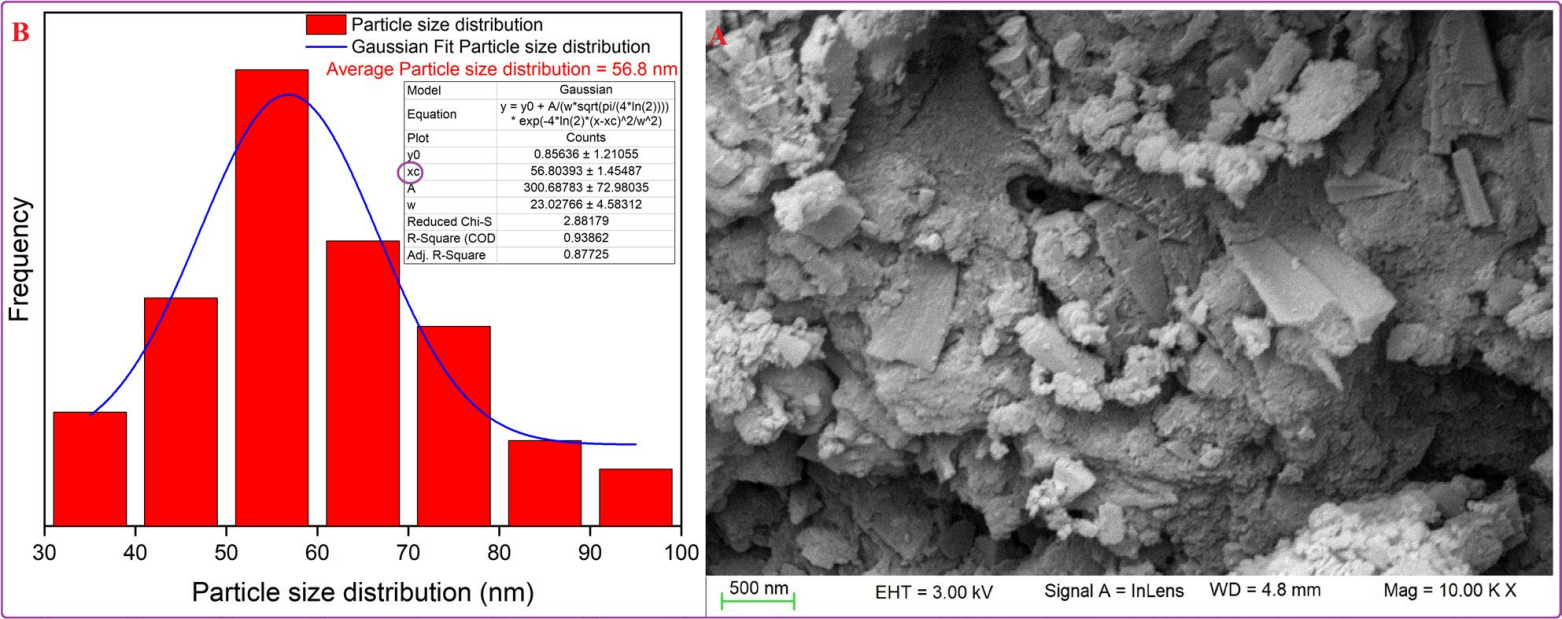
SEM (**A**), particle distribution (**B**) of the nano-synthesized mullite

Figure 6A reveals the SEM image of mullite nanoparticles, showing the porous morphology, irregular shape, and symmetric matrix. In addition, the Gaussian mixture model presented the particle size distribution and histogram in Fig. 6B using ImageJ (1.53e). The prepared nano mullite had an average particle size of 56.8 nm [34].

#### Transmission electron microscopy (TEM) of nano mullite

**Fig. 7:**
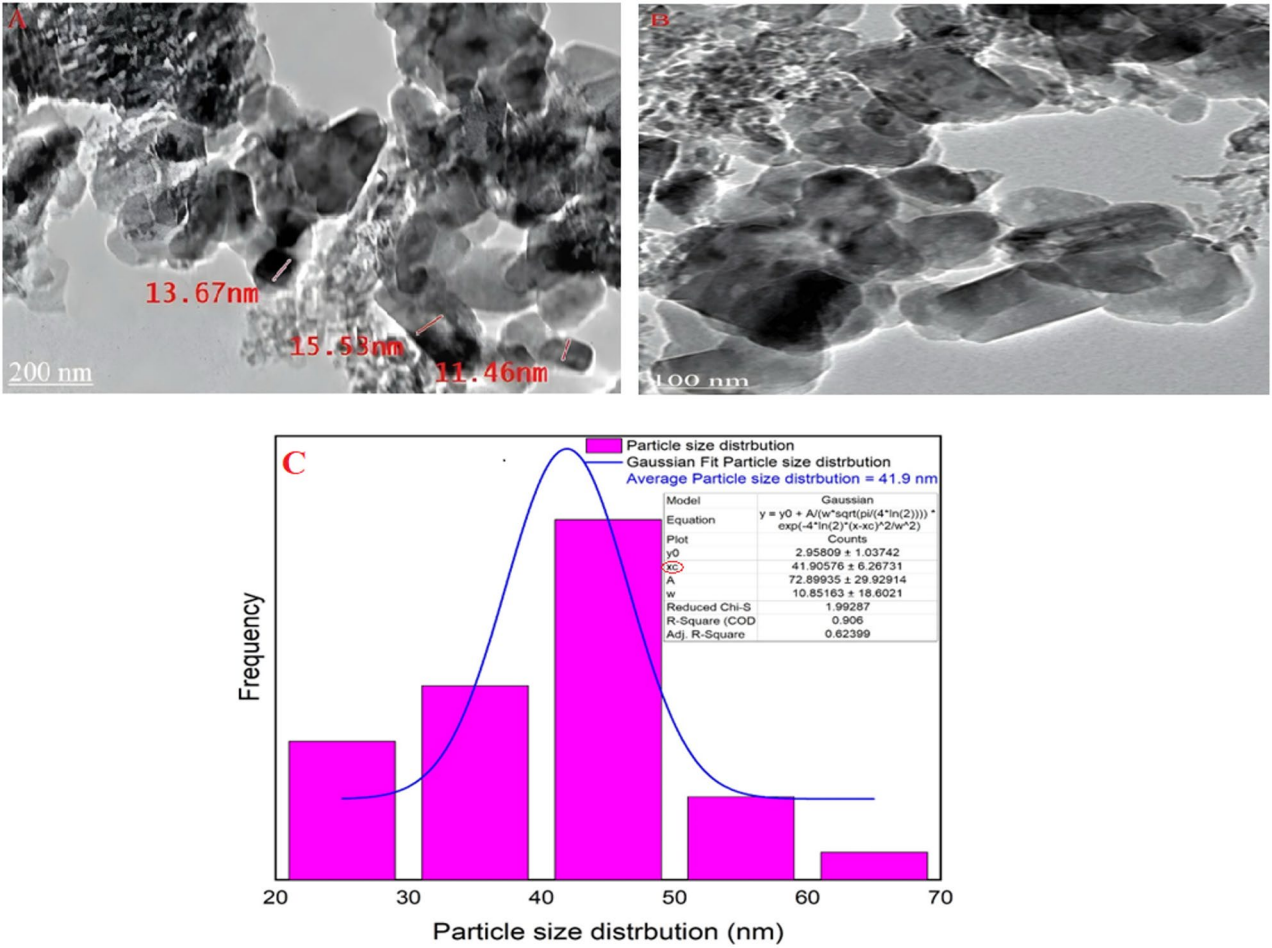
**A**, **B** The HR-TEM image and **C** particle size distribution of the synthesized nano mullite

As exhibited in Fig. 7A, B, the surface properties, crystalline characteristics, and particle size of mullite nanoparticles were displayed in (HR-TEM). Moreover, the orthorhombic crystal structure was recognized, and the particle size range of mullite nanopowder was found to be from 11.46 to 15.53 nm, consistent with an average crystal structure size of 7.46 nm from XRD analyses. It is important to recognize that variations between Transmission Electron Microscopy (TEM) and X-ray Diffraction (XRD) results are anticipated, as certain particles may comprise multiple crystallites [42, 43].

A sign of the substantial extent of crystallinity present in the material might be seen in the (HR-TEM) image of nano mullite, which clearly showed an ordered lattice edge structure. Moreover, the Gaussian mixture model presented the particle size distribution and histogram in Fig. 7C using ImageJ (1.53e). It was found that the average particle size is 41.9 nm [33].

#### Brunauer-Emmett-Teller (BET) analysis of mullite

**Fig. 8:**
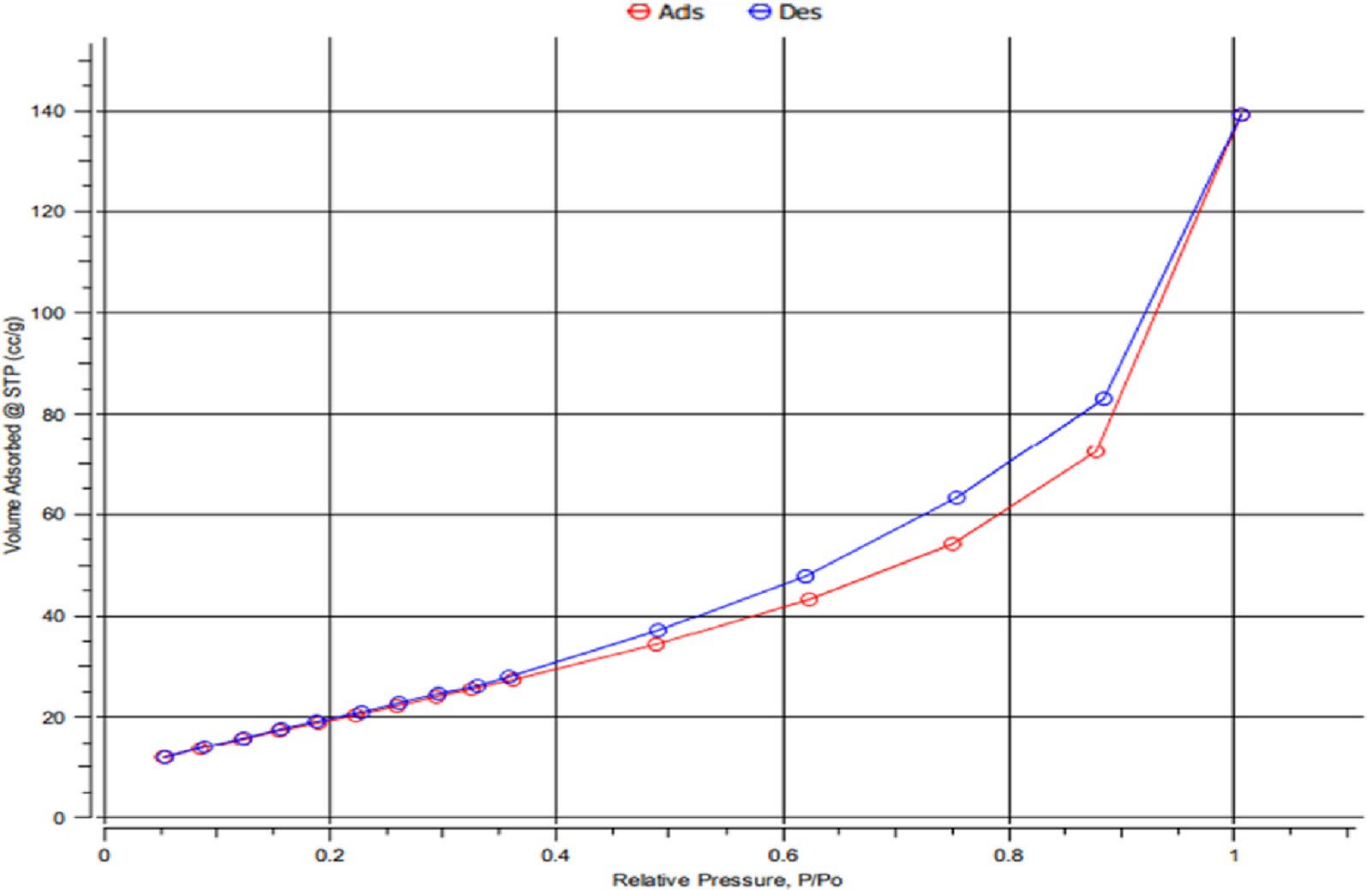
N_2_ adsorption–desorption isotherms for nano mullite

According to the nitrogen adsorption-desorption isothermal curve, it was discovered that the synthesized nano mullite had a hysteresis loop of type IV-(a)-H3 and the most significant amount of adsorbed volume was at P/P0 = 0.88 as shown in Fig. 8. Surface area, the total pore volume, and the average pore size of nano mullite were verified according to the BET analysis as 98.54 m²/g, 0.2254 cm^3^/g, and 4.9985 nm, signifying that they had a high surface area with a mesoporous structure, respectively.

### Characterization of the prepared nano t-ZrO_2_

#### XRD of the synthesized nano t-ZrO_2_

**Fig. 9:**
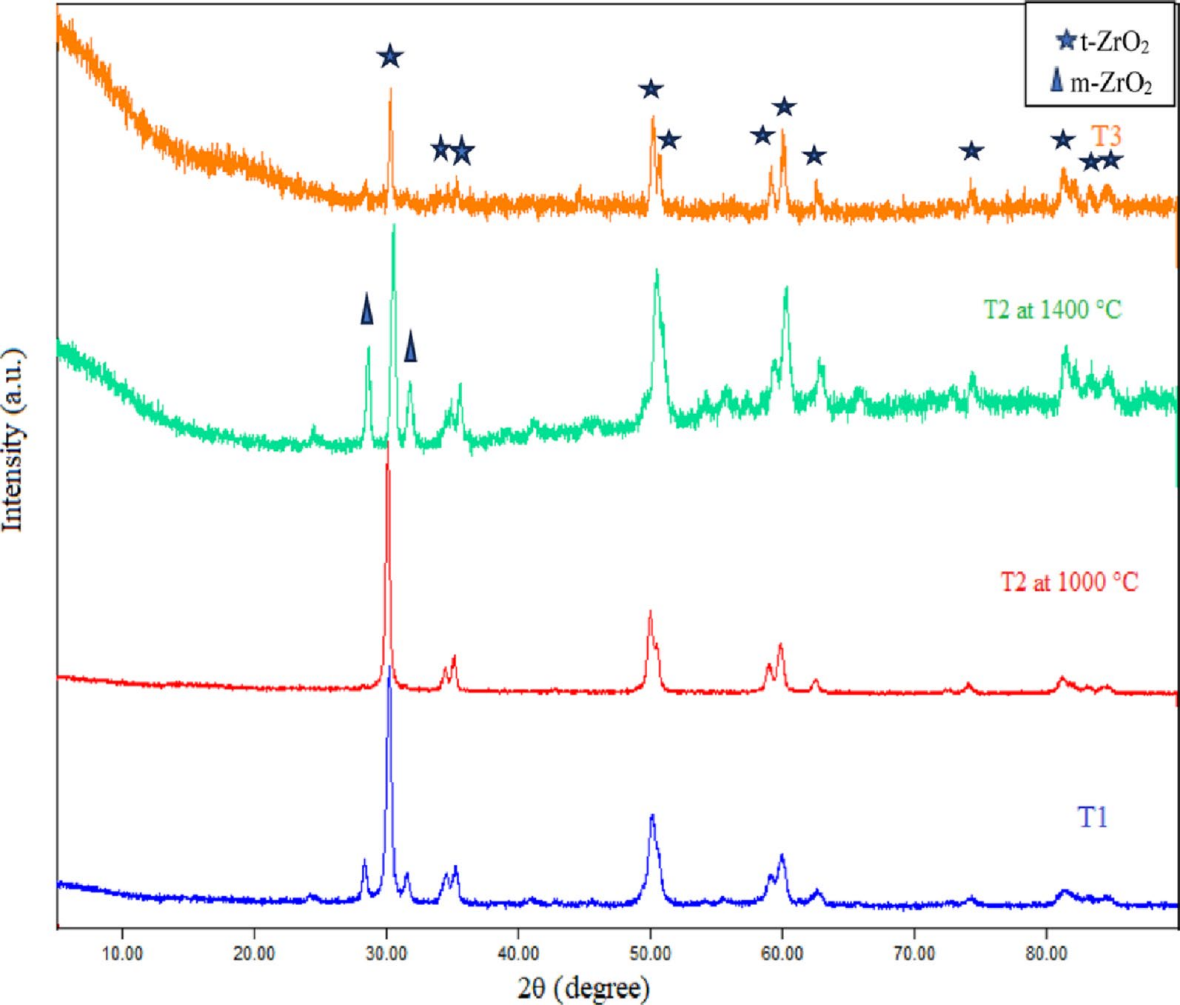
XRD patterns of nano ZrO_2_ for T1: 12% CeO_2_ at 1000 °C, T2: 14% CeO_2_ at 1000 °C, T2: 14% CeO_2_ at 1400 °C, T3: 15% CeO_2_ at 1400 °C

Figure 9 revealed the patterns in XRD of various zirconia nanoparticles doped with different amounts of ceria as a stabilizer at different temperatures (1000 °C and 1400 °C). The XRD patterns and Table 2 showed that sample T1, which contained 12% CeO_2_ at 1000 °C, had a monoclinic and tetragonal phase of zirconia by percentages of 19.9% and 80.1%, respectively; however, sample T2, which contained 14% CeO_2_ at the same firing temperature (1000 °C) showed a pure tetragonal phase. Sample T2 produced a pure tetragonal phase of zirconia at 1000 °C. To ensure that sintering at temperatures above 1000 °C would not cause a transition from the tetragonal to the monoclinic phase, sample T2 was fired at 1400 °C and then examined by XRD. The XRD pattern for T2 at 1400 °C showed the appearance of a monoclinic phase beside the tetragonal phase in a ratio of 40.9% to 59.1%, respectively (Table 2). So, sample T3, which contained 15% CeO_2_ at 1400 °C, was examined by XRD and showed a pure tetragonal phase of zirconia. Consequently, T3 was the most suitable sample containing pure t-ZrO_2_ to be exploited in the synthesis of the porous ceramic composites. Successively, the peaks of the XRD patterns of sample T3 were sharp and definite peaks, which revealed significant levels of crystallinity of the prepared t-ZrO_2,_ and the average crystallite size was 29.16 nm (as stated by the Debye-Scherrer equation) [35, 44]. As well as the space group of metastable tetragonal zirconia was P4₂/nmc (No. 137), and the diffraction peaks 2θ at 29.98°, 34.32°, 44.23°, 49.80°, 58.78°, 59.81°, and 62.29° were assigned to (011), (002), (012), (123), (013), (121), and (022) planes respectively according to card.No.: JCPDS 50-1089 [45, 46].

**Table 2:** The percentages of monoclinic and tetragonal phases in several zirconia nano samples with the amount of used ceria as well as the calcined temperature and the average crystallite size (according to XRD analyses)

Samples	Ceria percentage (%)	Calcination temperature (°C)	Monoclinic zirconia	Tetragonal zirconia (%)	Average crystallite size (nm)
T1	12	1000	19.9%	80.1	18.2
T2	14	1000	–	100	22.4
	14	1400	40.9%	59.1	26
T3	15	1400	–	100	29.16

#### Scanning electron microscope (SEM) of t-ZrO_2_ nanoparticles

**Fig. 10:**
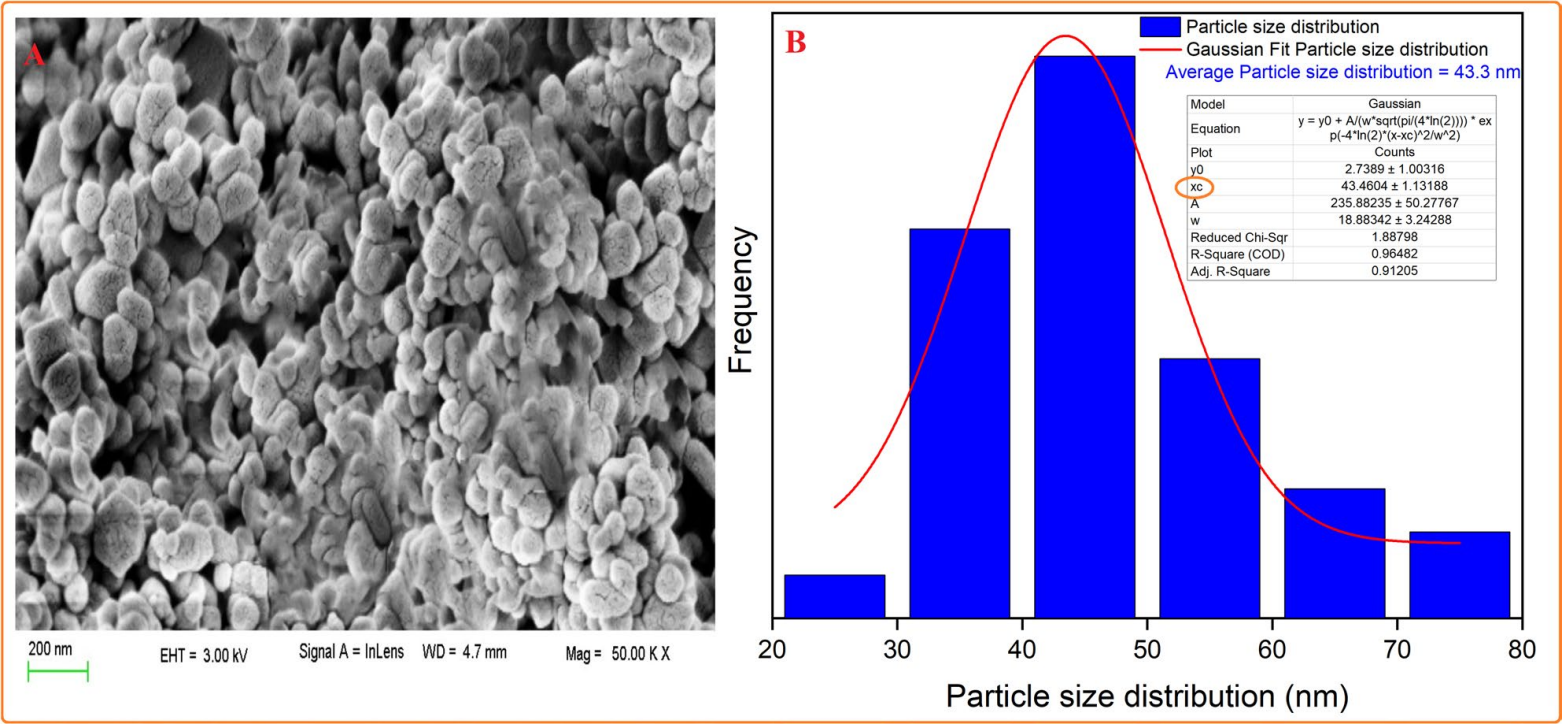
SEM (**A**) and particle distribution (**B**) of the nano-synthesized t-ZrO_2_ nanoparticles

Figure 10A depicts a SEM picture of t-ZrO_2_ nanoparticles, revealing their porous morphology, uniform matrix, and appearing as white spots. Moreover, the Gaussian mixture model used ImageJ (1.53e) to display the particle size distribution and histogram in Fig. 10B. The produced nano t-ZrO_2_ particles had an average size of 43.46 nm [35].

#### Transmission electron microscope (TEM) of nano t-ZrO_2_

**Fig. 11:**
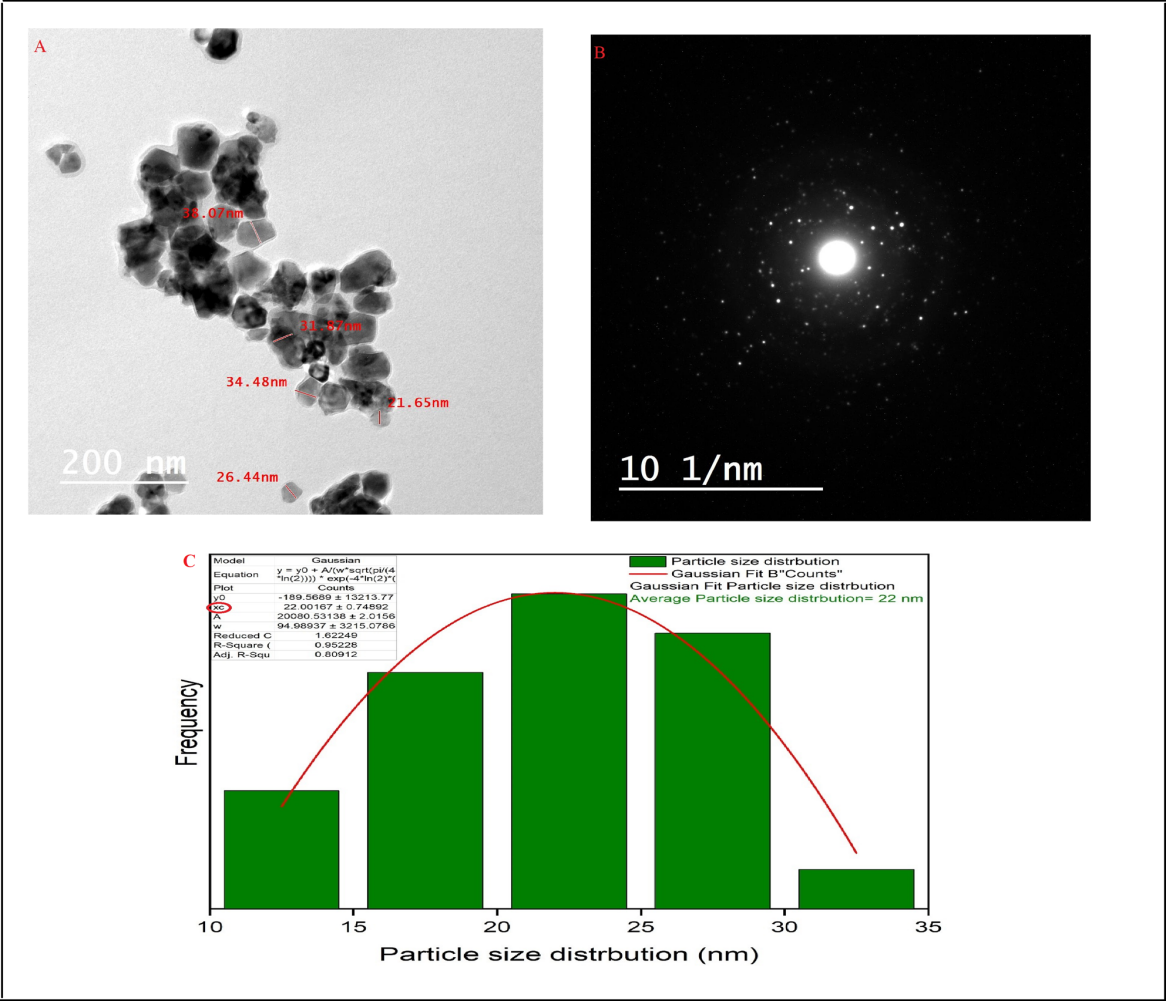
**A** The HR-TEM, **B** SAED, and **C** particle size distribution of the synthesized t-ZrO_2_ nanoparticles

As shown in Fig. 11A, B, TEM images were used to analyze the particle size of the synthesized nano t-ZrO_2_ and revealed that the average size of particles was in the range from 21.65 to 38.07 nm, which agreed with the average crystallite size calculated from XRD analysis, which was 29.16 nm. Overall analyses proved that the prepared t-ZrO_2_ was on a nanoscale. The straightforward, practical lattice edge configurations observed in (HR-TEM) of zirconia nanoparticles, along with (SAED), revealed the extraordinary crystal structure of the prepared t-ZrO_2_ nanoparticles, as illustrated in Fig. 11A, B [35, 47]. Furthermore, the particle size distribution was presented by the Gaussian mixture model and histogram in Fig. 11C using ImageJ (1.53e). It was found that the average particle size is 22 nm [33].

**Fig. 12:**
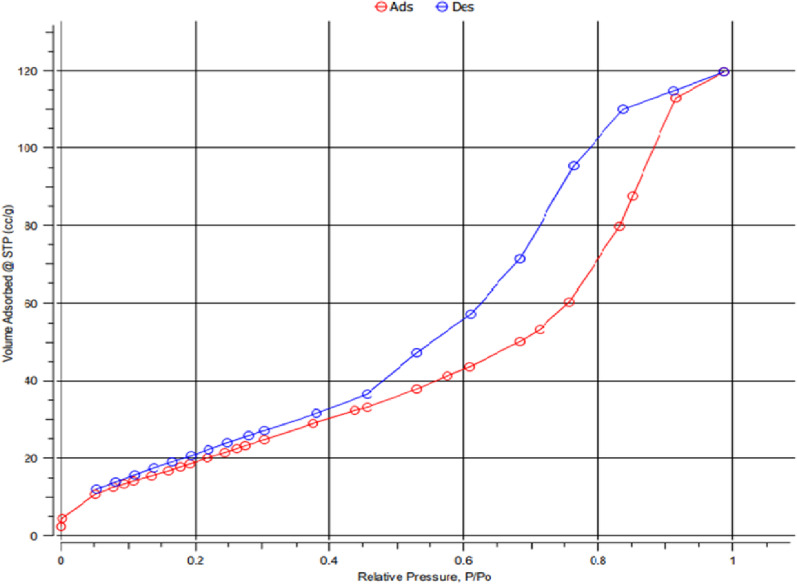
N_2_ adsorption–desorption isotherms for nano t-ZrO_2_

**Fig. 13:**
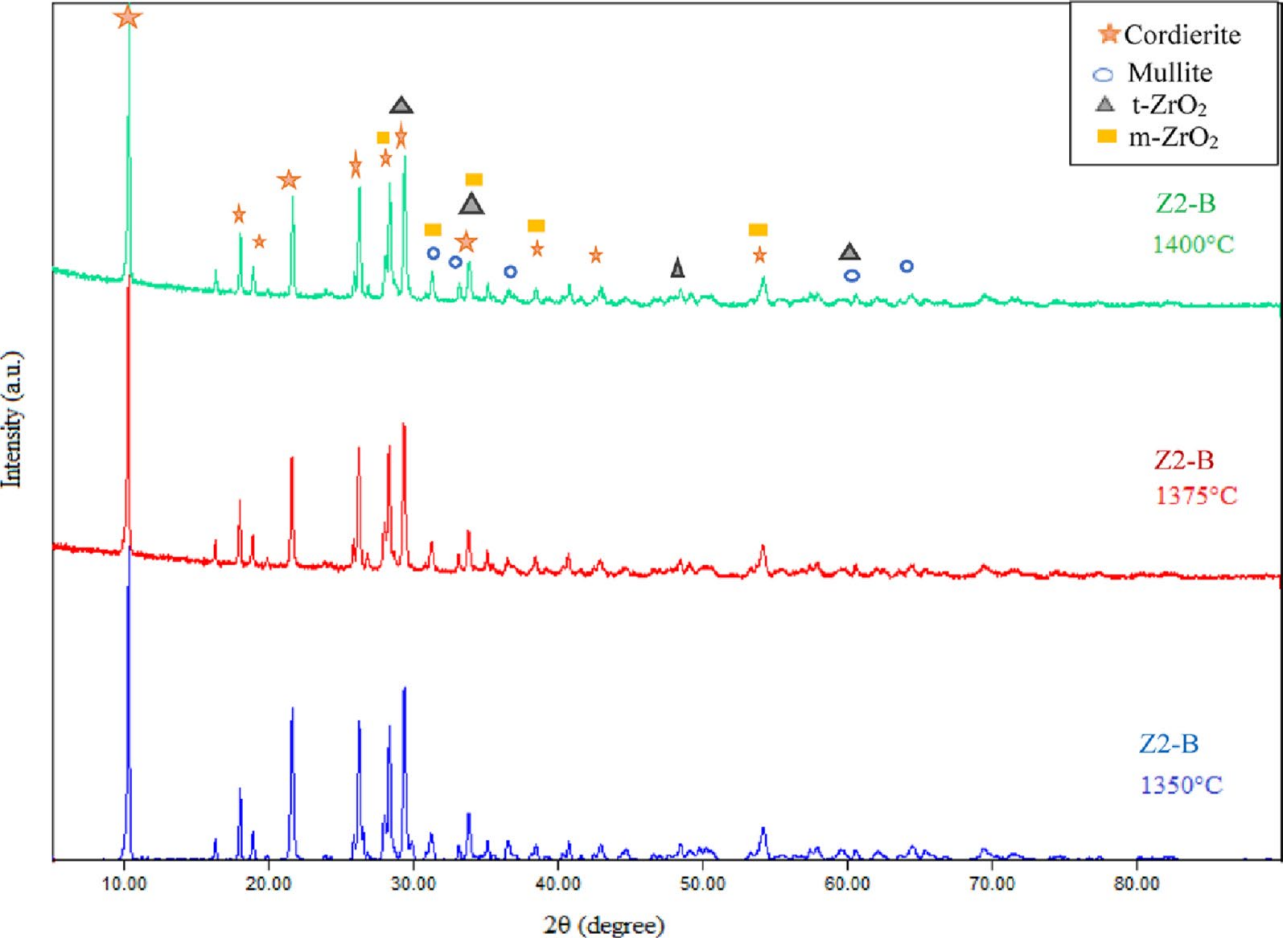
The phase composition by XRD patterns of the sintered composite (Z2-B) containing bagasse ash which was sintered at 1350 °C, 1375 °C and 1400 °C

#### Brunauer–Emmett–Teller (BET) analysis

The nitrogen adsorption-desorption isothermal curve for the synthesized nano t-ZrO_2_ revealed that the isothermal curve had a hysteresis loop of type IV(a) and reached the maximum adsorbed volume at P/P_0_ = 0.99 as demonstrated in Fig. 12. The BET analysis also demonstrated the surface area of nano t-ZrO_2_ was 80.99 m²/g as well as the pore volume of about 0.178 cm^3^ /g with an average pore size of 5.121 nm, which confirmed the mesoporous structure of the nano tetragonal zirconia [48].

### Characterization of the cordierite–mullite–zirconia porous ceramic

#### Phase composition

**Fig. 14:**
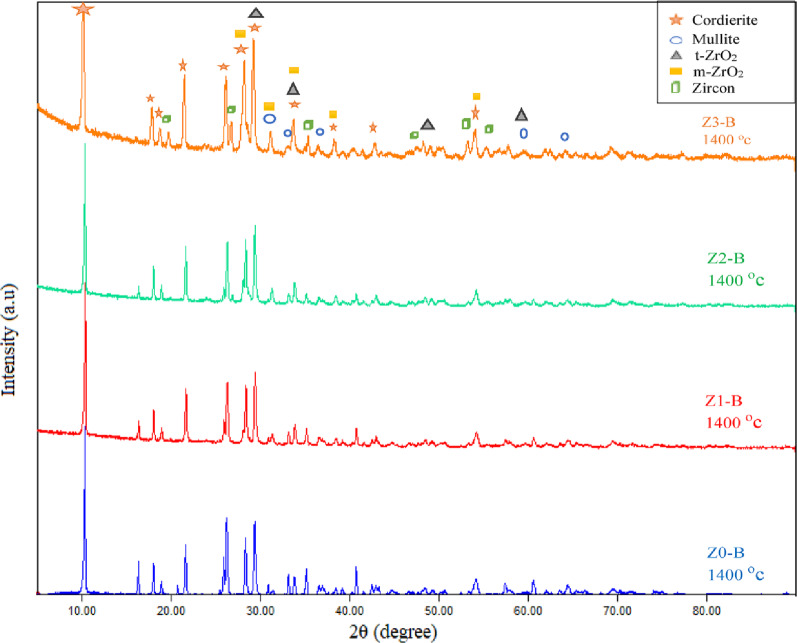
The phase composition by XRD patterns of the sintered composites (Z0-B, Z1-B, Z2-B, Z3-B) containing bagasse ash which were sintered at 1400 °C

**Fig. 15:**
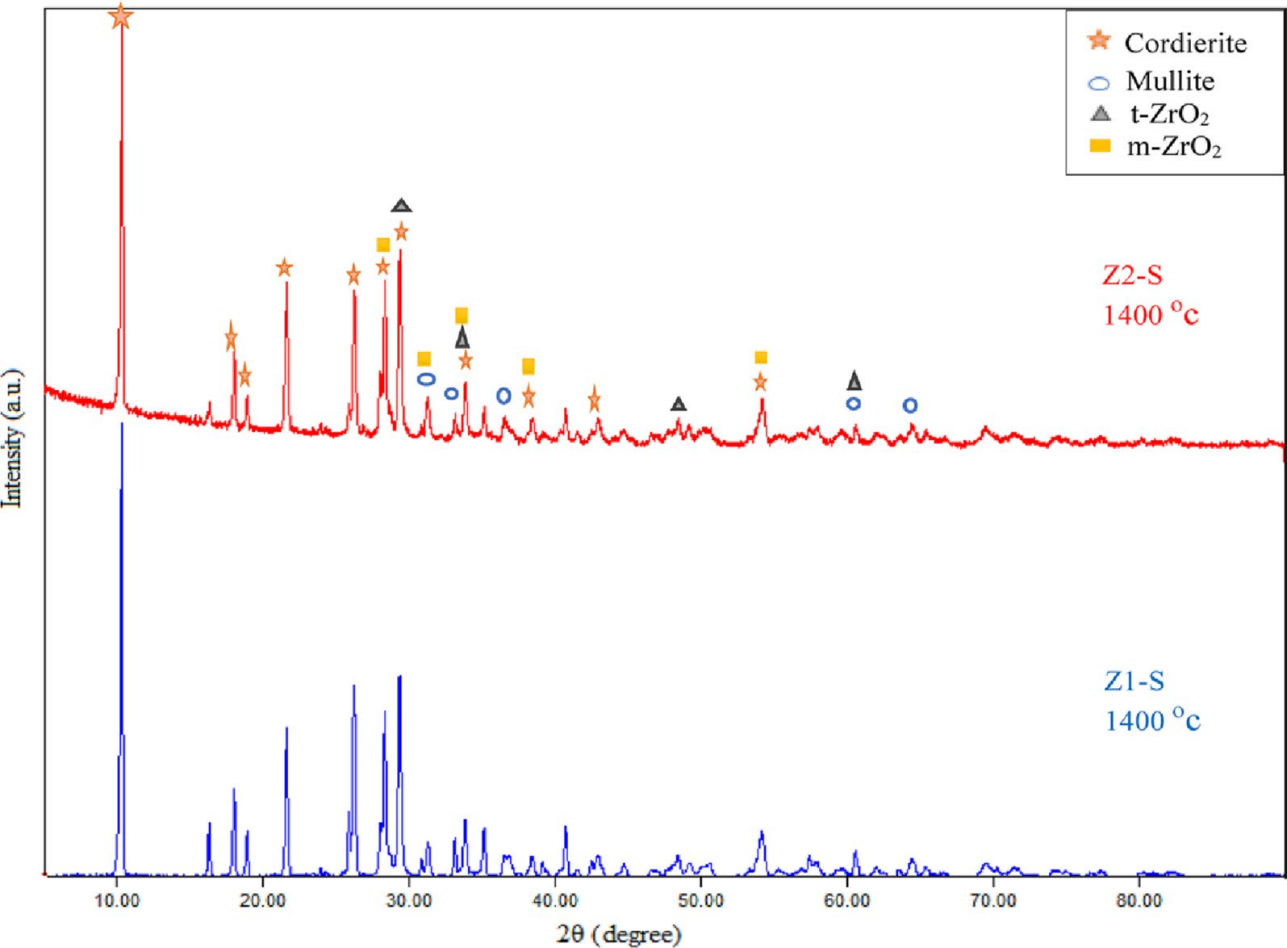
The phase composition by XRD patterns of the composites (Z1-S) and (Z2-S) containing sawdust ash sintered at 1400 °C

By using the XRD technique to determine the phase composition of the porous ceramic materials, Figure 13 showed the phase composition of a sintered sample (Z2-B) containing bagasse ash fired at different temperatures of 1350 °C, 1375 °C, and 1400 °C. The main phases observed in the sample (Z2-B) at all three sintering temperatures are cordierite (Mg_2_Al_4_Si_5_O_18_), mullite (Al_6_Si_2_O_13_), m-zirconia (ZrO_2_) and t-zirconia (ZrO_2_). The cordierite was confirmed according to to JCPDS / PDF Card No.: 13–0294, space group of Cccm (66) with an orthorhombic crystal system, and the specified diffraction peaks 2θ of cordierite were 10.39°, 18.07°, 19.00°, 21.91°, 26.47°, 28.34°, 29.58°, 33.94°, 38.50°, and 54.27° which correlated with (200), (310), (002), (112), (022), (402), (131), (422), (240), and (624) planes, respectively. Moreover, The diffraction peaks 2-theta of the mullite phase were 31.50°, 33.26°, 35.23°, 40.83°, 60.52°, and 65.59 ° attributed to planes of (001), (220), (111), (121), (340), and (250), respectively. According to card number **JCPDS 15-0776**, the space group was Pbam (No. 55), and also the crystal system of the mullite phase was orthorhombic. The tetragonal and monoclinic phases of zirconia were observed in all sintering temperatures. The diffraction peaks which confirmed the appearance of the tetragonal phase of zirconia were 2θ = 29.54°, 34.10°, 43.01°, 50.06°, 59.59°, and 62.08° with indexed planes (011), (002), (012), (020), (121), and (022), respectively, according to card number JCPDS 50-1089 and space group of P4₂/nmc (No. 137). Whereas the zirconia monoclinic phase’s diffraction peaks were 2θ = 28.19°, 31.40°, 35.45°, 38.45°, and 54.10° with planes of (111), (111), (200), ( 012), and (202), respectively and fit to P 1 21/c 1 (14) space group based on card number 2300296. The same phases composite also appeared in the samples (Z0-B, Z1-B, Z2-B) containing bagasse ash, which were sintered at 1400 °C (Fig. 14). The monoclinic phase of zirconia in the samples after sintering may be due to the mismatch between zirconia’s and cordierite’s thermal expansion coefficients. As mentioned, cordierite has a low value of thermal expansion coefficient ( (1–2) × 10^− 6^ °C ^− 1^ ) in contrast to the value of zirconia (9.7 × 10^− 6^ °C ^− 1^), it is probably creating tensile stress on t-ZrO_2_ particles which motivates the transformation into m-ZrO_2_ [49]. It was discovered that with a high content of zirconia (25 wt%), the zircon (ZrSiO_4_) phase was formed because of the high affinity of zirconium ions to react with silica at high sintering temperatures (more than 1200 °C) [50, 51]. So, zircon peaks were observed in the (Z3-B) sample containing 70 wt% cordierite and 30 wt% zirconia sintered at 1400 °C, as shown in Figure (14). According to 9,002,561, the space group of zircon was I 41/a m d (141), and the crystal structure was tetragonal. Additionally, the diffraction peaks of zircon at 2θ = 20.03°, 26.87°, 34.02°, 35.68°, 38.58°, 40.76°, 43.76°, 47.70°, 53.50°, 55.58° which were correlated to (101), (200), (211), (112), (220), (202), (301), (013), (312), (213) planes, respectively. The enhancement of α-cordierite crystallinity peaks and the disappearance of the residual phase peaks like the spinel (MgAl_2_O_4_) phase is also noticeable in each sintering temperature. This is ascribed to the presence of ceria, which not only stabilizes the t-ZrO_2_ but also prevents the formation of any unwanted phases [51, 52]. Additionally, during cordierite sintering, this phase formation prevented the development of undesirable spinel phases and encourages the crystallization of the cordierite phase. This effect enhances sinterability and improves the physical, mechanical, and electrical properties of the resulting ceramics, especially in samples containing bagasse ash, because it is present in about 4.40 wt% [53, 54]. Also, the XRD pattern of the samples that have sawdust ash and sintering at 1400 °C, such as (Z1-S) and (Z2-S), have the same trend and phases that appeared in the samples of the bagasse ash (Fig. 15).

### Densification parameters (BD and AP)

**Fig. 16:**
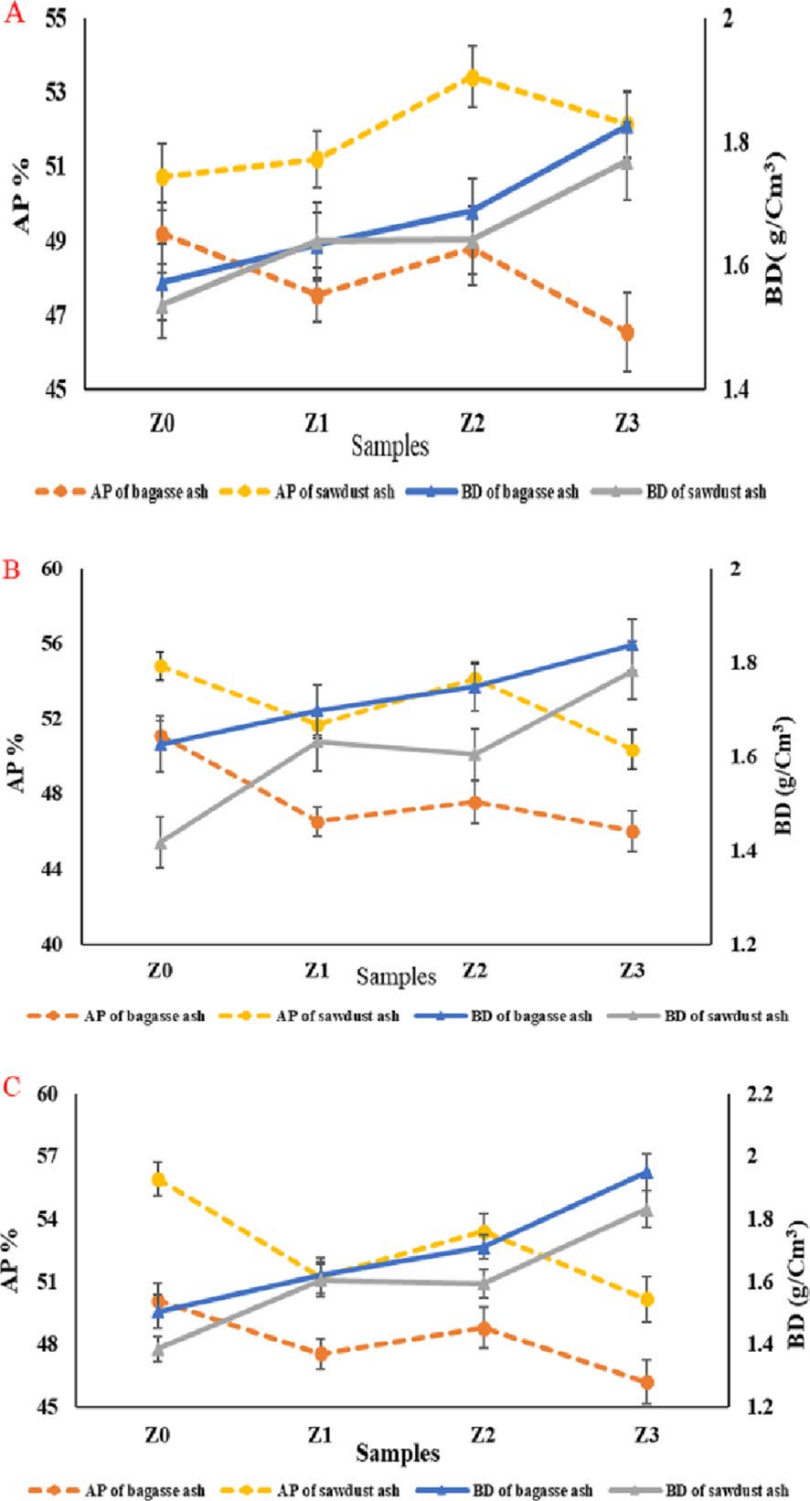
A–C The BD and AP of the sintered samples (Z0-Z3) containing sawdust and bagasse ash at 1350 °C,1375 °C, and 1400 °C, respectively

According to Archimedes’ method, the sintered samples’ bulk density and apparent porosity were measured. As displayed in Fig. 16A–C, the BD and AP of the sintered composites containing bagasse ash (Z0-B)-(Z3-B) and sawdust ash (Z0-S)-(Z3-S) at several temperatures of 1350 °C, 1375 °C, and 1400 °C, respectively, were studied. It was discovered from the BD and AP results that with increasing the sintering temperatures, BD increased, and AP decreased. This occurred owing to the process of solid-state sintering, which led to the grain growth and diffusion of particles from boundaries to voids and cavities, resulting in dense samples [55]. Furthermore, the development in BD of the samples in Fig. 16A–C with increasing nano zirconia content at the same sintering temperature was observed due to an increase in density of tetragonal zirconia phase (6.04 g/cm^3^) than cordierite phase (2.53 g/cm^3^), which influenced positively the densification of the samples [56, 57]. The same trend was observed in all sintering temperatures, and specifically, the sintering temperature of 1400 °C showed the best improvements in BD. While the samples containing sawdust ash exhibited the highest AP values, which was attributed to the high carbon content of sawdust ash more than bagasse ash, as indicated in Supplementary Table 1. The carbon has oxidized to form carbon mono and dioxides, leaving empty pores and cavities during high-temperature sintering, resulting in a considerable increase in sample porosity [13]. It was also found that sample Z3 containing bagasse or sawdust ash significantly increased BD and decreased AP at all sintering temperatures. This is due to the formation of a zircon phase, which improved the solid-state reaction mechanism between the phases in the sample and increased its densification [58]. Also, SO_3_ in ash is a very small percentage compared to the high carbon percentage in ashes. So, the main concern is the carbon percentage. In point of fact, the presence of Fe₂O₃ may impact the sintering behaviour by enhancing the sintering, which is already used as a sintering aid in different sintering processes [54].

Consequently, sample Z2 containing bagasse ash or sawdust ash sintered at 1400 °C was considered the best composite because its BD and AP values were 1.7–1.6 g/cm^3^ and 48.8–53.4%, respectively.

### Linear change

**Fig. 17:**
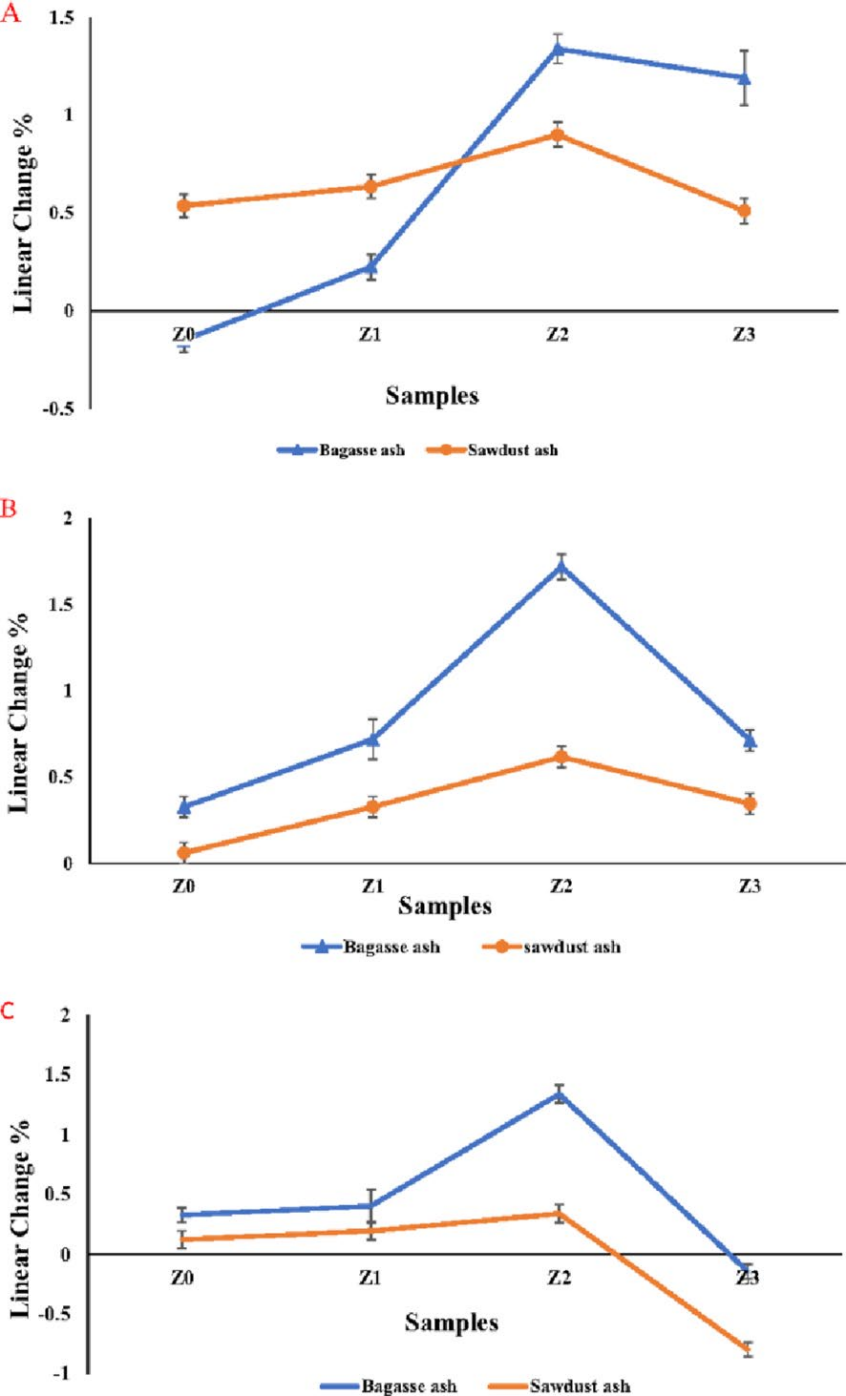
**A**–**C** Sawdust ash and bagasse ash samples’ linear change (Z0-Z3) at (**A**) 1350 °C, (**B**) 1375 °C, and (**C**) 1400 °C

Figure 17A–C displayed the samples’ linear change with a pore-forming agent (sawdust ash or bagasse ash) at different sintering temperatures of 1350 °C, 1375 °C, and 1400 °C, respectively. It was observed that the samples shrank as the sintering temperature increased. This is because of the method known as solid-state sintering [4]. On the other hand, it was discovered that the presence of bagasse ash or sawdust ash inhibited the sintering in samples causing samples to expand. Also, the specimens’ cavities and apparent pores make the sintering process more challenging [13]. In addition, the tetragonal (t) to monoclinic (m) phase transformation of zirconia was associated with a volume expansion of about 4–5%, leading to sample expansions [59].

### The cold crushing strength (CCS)

**Fig. 18:**
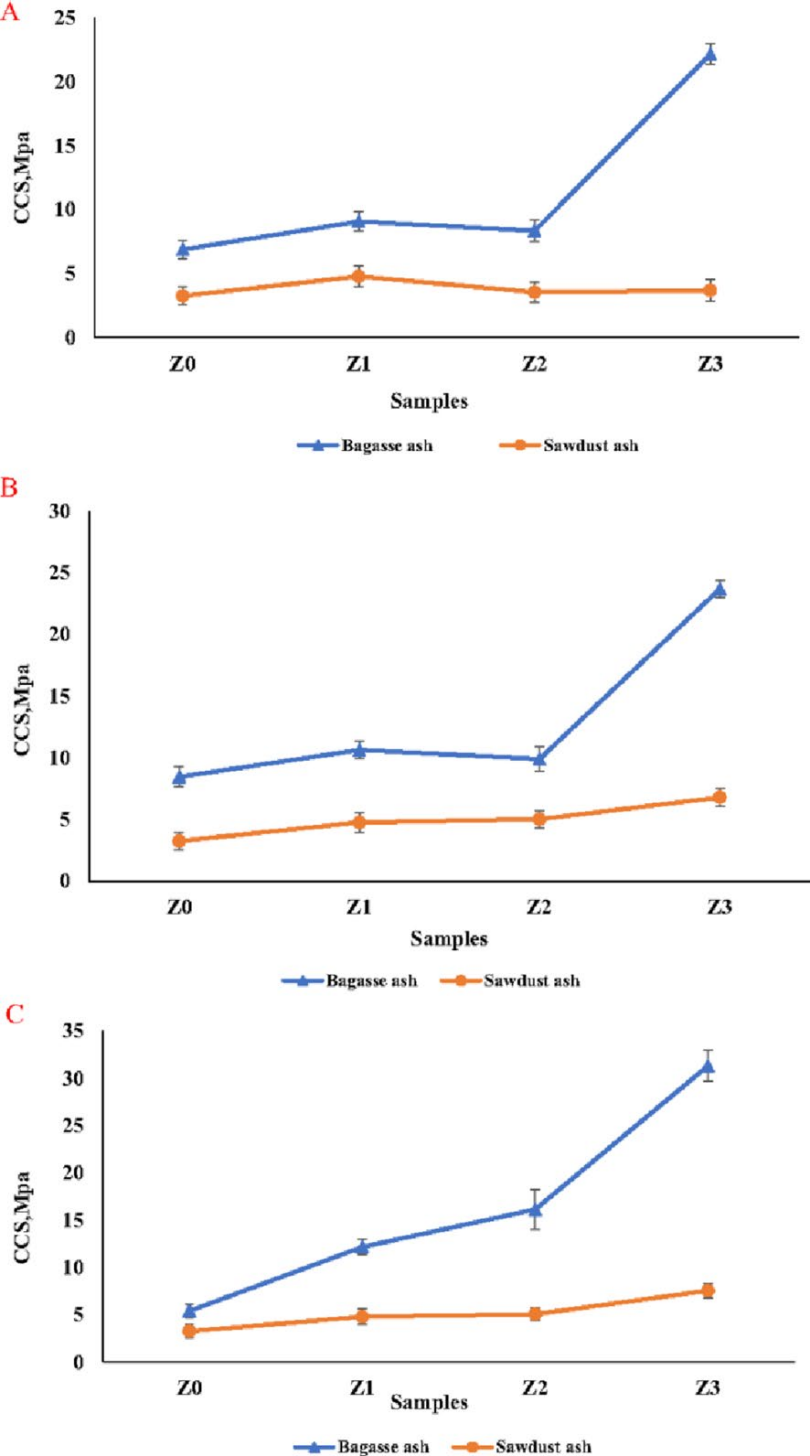
**A**–**C** The CCS of the sintered samples (Z0-Z3) containing the bagasse ash and sawdust ash at different sintering temperatures of 1350 °C, 1375 °C, and 1400 °C, respectively

As exhibited in Figs. 18A–C, the CCS of the sintered samples (Z0-Z3) containing different pore-forming agents (bagasse ash or sawdust ash) at temperatures of 1350 °C, 1375 °C, and 1400 °C, respectively. It was discovered that the CCS was improved with increasing the sintering temperatures. This is because the solid-state sintering process had been improved by raising the sintering temperatures to reach 1400 °C, which was also confirmed by the linear change results, which showed the shrinkage of samples with temperature increase (Fig. 17). Furthermore, the increase in CCS from sample Z0 to sample Z3 could be attributed to an increase in nano t-ZrO_2_ additions, which enhanced the samples’ mechanical qualities. This is related to zirconia’s tetragonal to monoclinic phase transformation, which is accompanied by a volume expansion of about 4–5%, stopping crack propagation and improving the mechanical qualities of the samples. It was also detected that the samples (Z0-B)-(Z3-B) containing bagasse ash as a pore-forming agent had higher CCS values than samples containing sawdust ash as a pore-forming agent. This could be verified by Supplementary Table 1, which showed the higher carbon content of sawdust ash than the bagasse ash, which increased the AP values and decreased the BD, resulting in the CCS decreasing, as shown in Fig. 16. It was also noticed that a significant improvement in the CCS of the sample Z3 containing bagasse ash (Z3-B) or sawdust ash (Z3-S) at all sintering temperatures of 1350 °C, 1375 °C and 1400 °C more than the other samples. This is due to the formation of the zircon phase in samples containing 30% of t-ZrO_2_ with high sintering temperatures exceeding 1200 °C, which increased the BD, leading to the considerable enhancement in the CCS, as proved in the XRD pattern in Fig. 14 and densification parameters in Fig. 16A–C. So, the samples (Z2-B) and (Z2-S), which were sintered at 1400 °C showed a good result in BD, AP, and CCS (1.7 g/cm^3^, 48.9%, and 16.14 MPa) and (1.59 g/cm^3^, 53.4%, and 5.06 MPa), respectively.

### Pore size distribution of the sintered samples

**Fig. 19:**
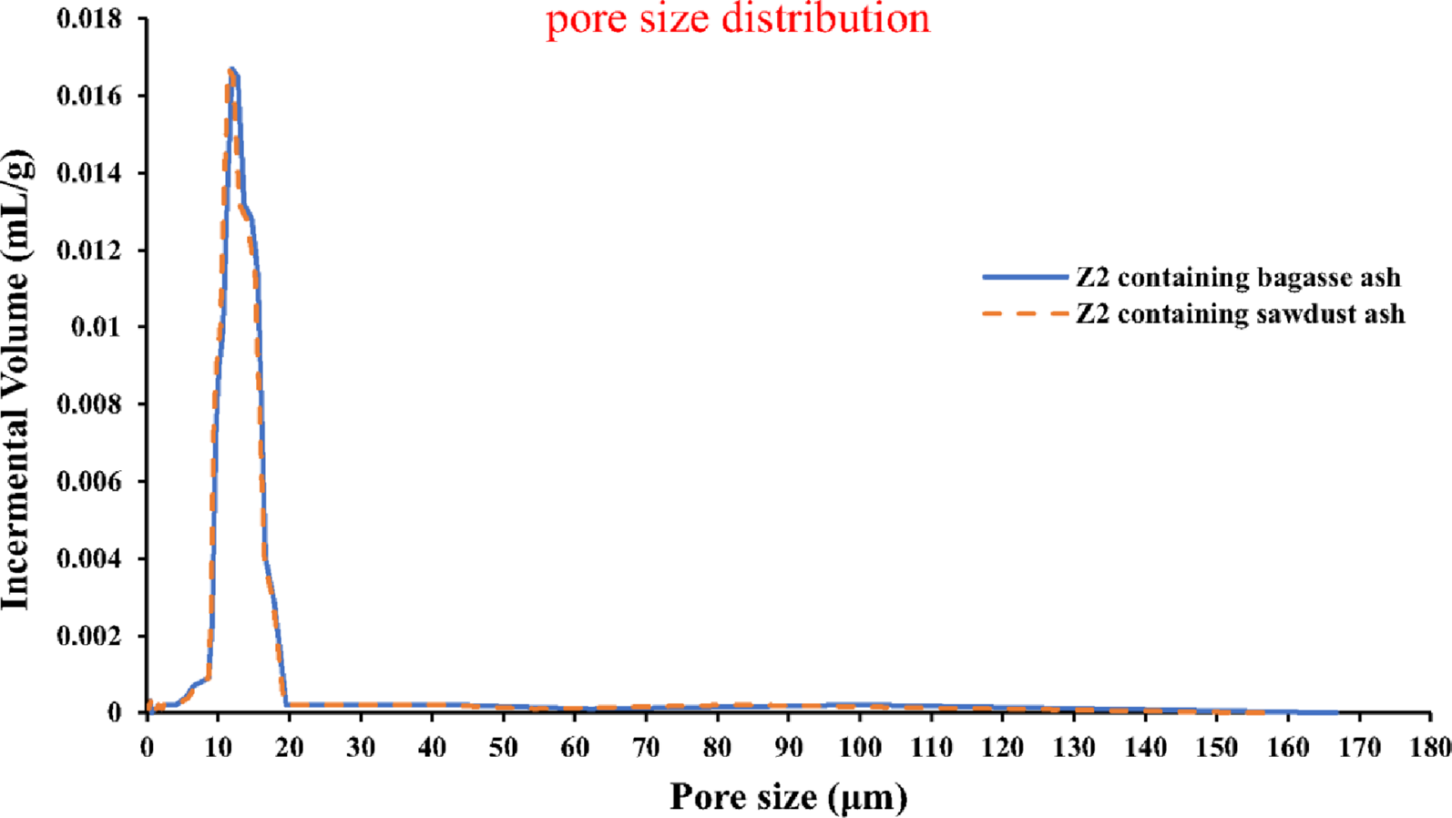
The pore size distribution of the porous samples (Z2-B) and (Z2-S) which were sintered at 1400 °C

As demonstrated in Fig. 19, the pore size distribution was estimated by a mercury porosimeter precisely in the samples (Z2-B) and (Z2-S) containing bagasse ash and sawdust ash, respectively [60, 61]. A sharp peak was observed from the curve of pore size distribution, which exhibited a consistent pore size. The average pore size of the sample (Z2-B) containing bagasse ash was 12.77 μm, and the pore size range was from 1.12 μm to 19.55 μm. While the average pore size of the sample (Z2-S) containing sawdust ash was 12.07 μm and the pore size ranged from 1.12 to 19.27 μm. The previous data revealed unimodal characteristics and similar pore sizes. Consequently, the sintered samples were recognized as macro-porous ceramics whose pores size was higher than 50 nm and smaller than 5 mm [6, 62]. These pores have been effectively utilized throughout numerous industrialized functions, including thermal insulators, lightweight structural elements, filtration, absorption, catalysts, and catalytic supports [63].

### Microstructure (SEM conjugated with EDAX)

**Fig. 20:**
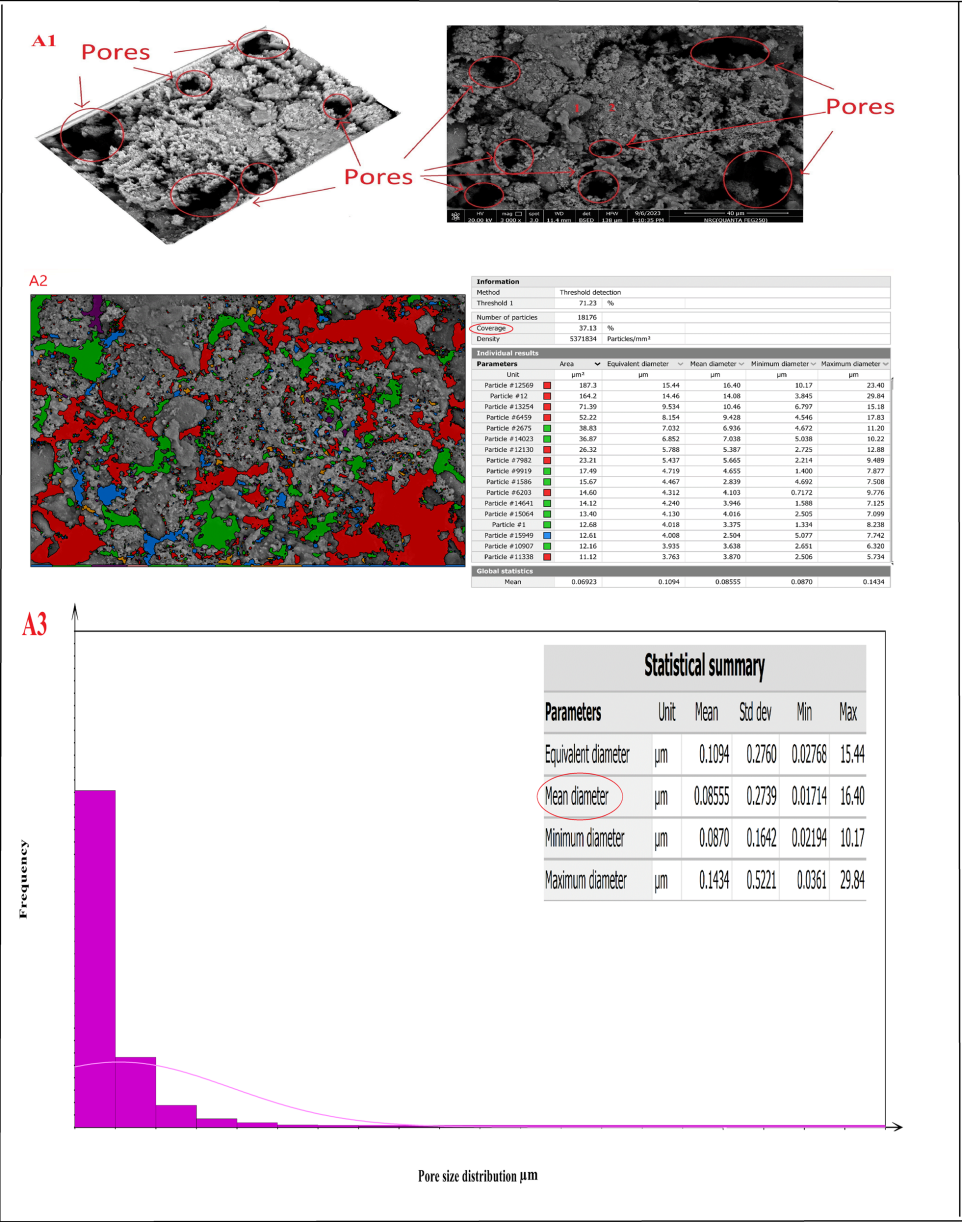

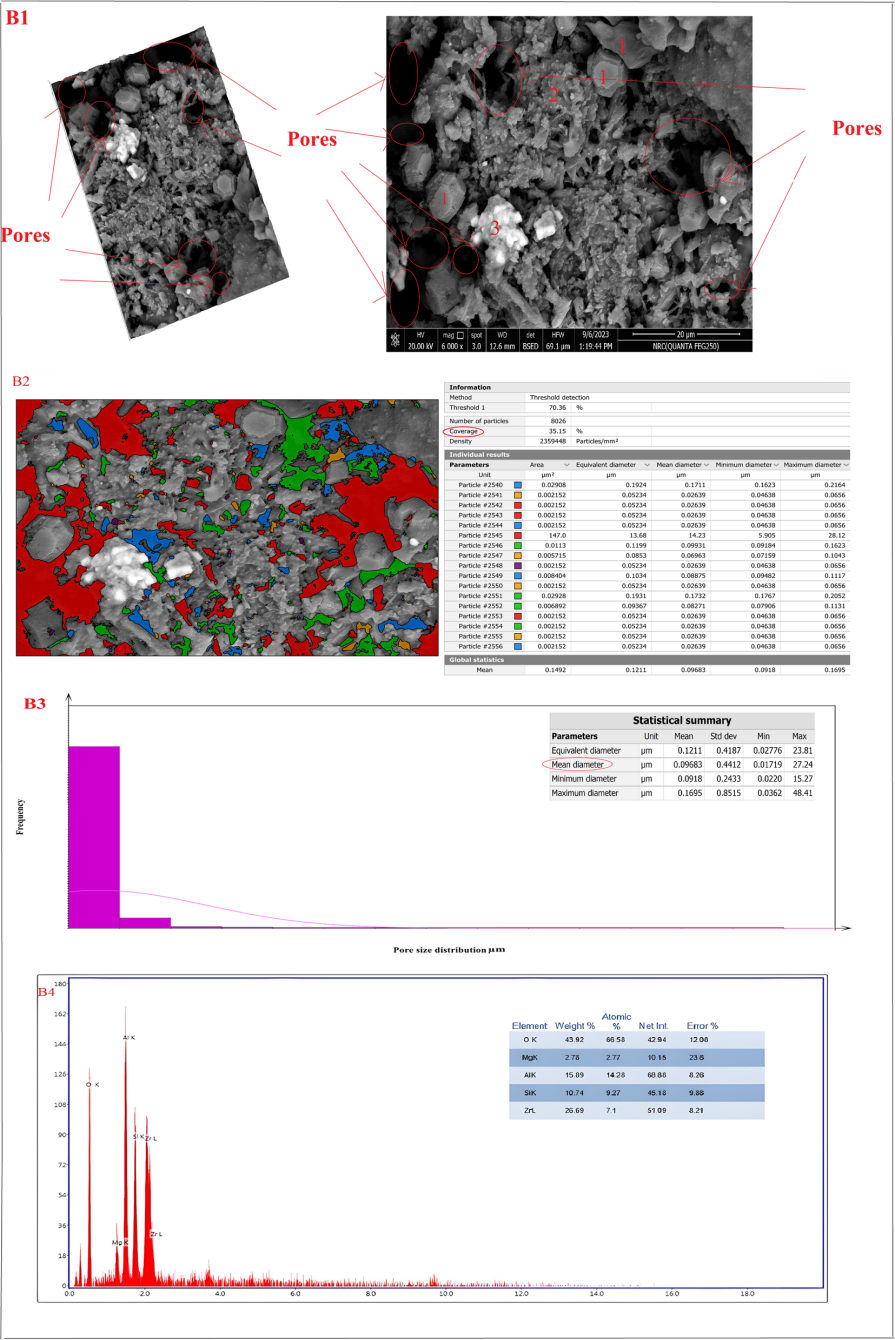

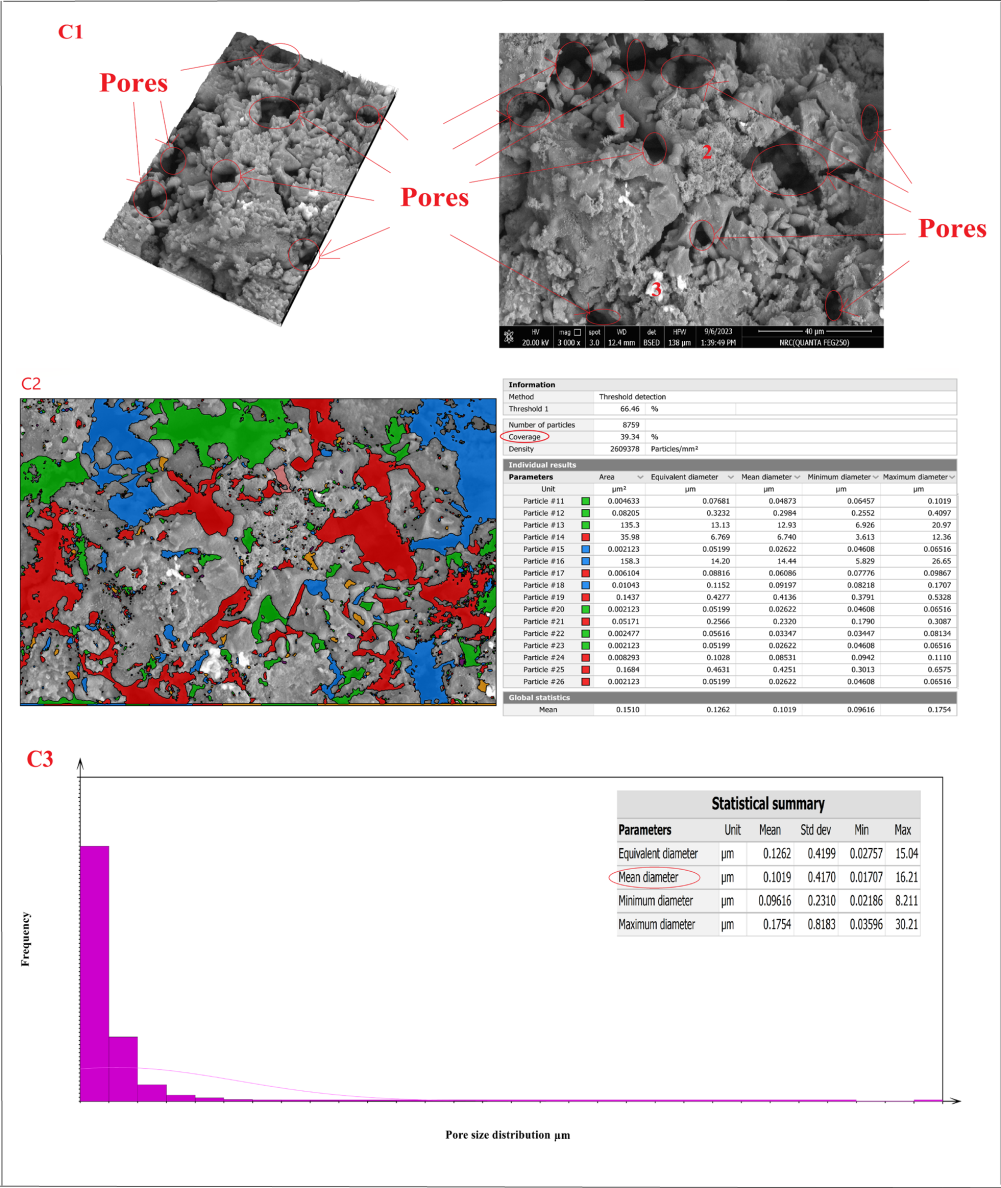

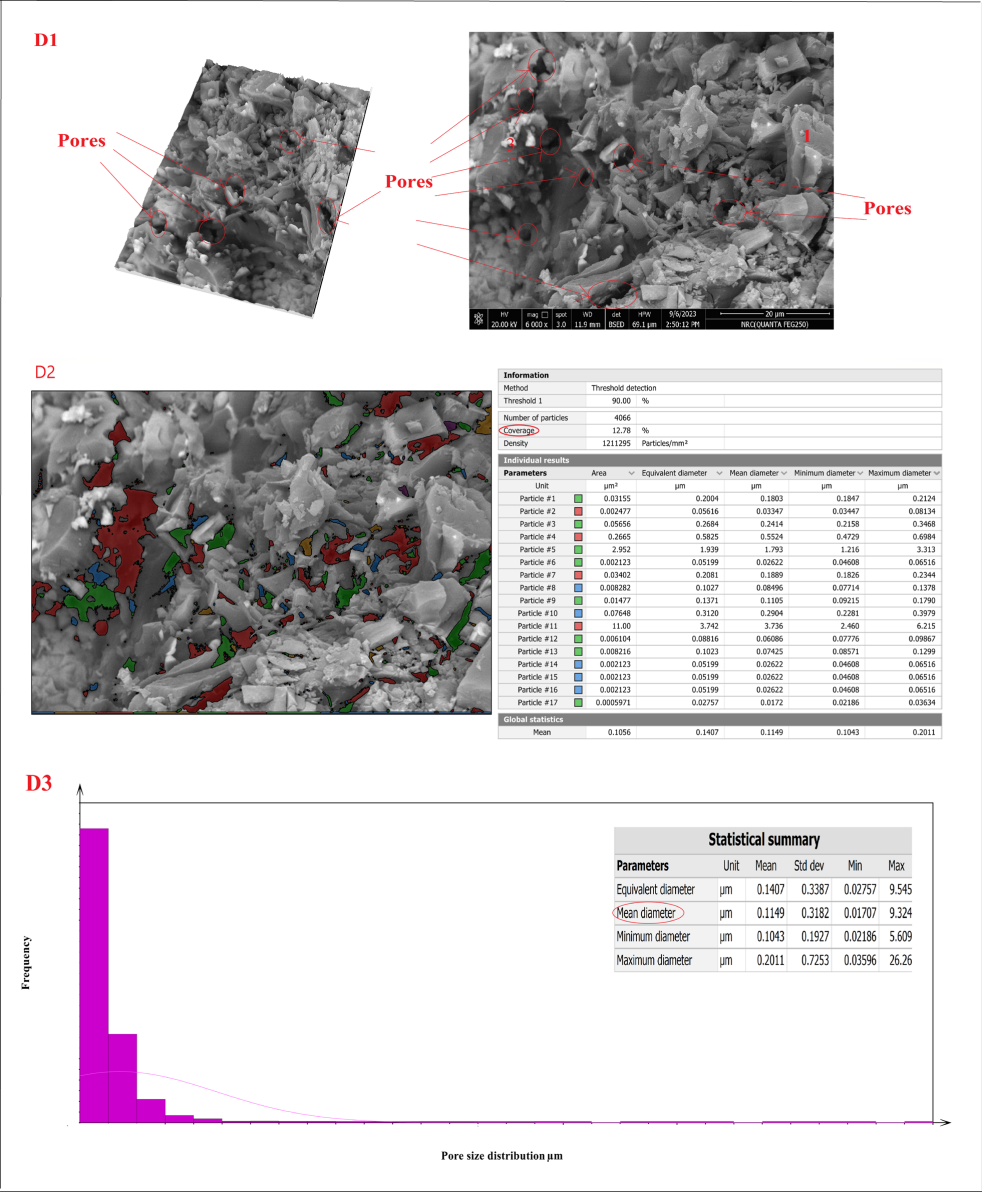
(**A1**–**A3**, **B1**–**B3**, **C1**–**C3**, **D1**–**D3**). SEM micro images, pore size distribution for sintered samples (Z0-B), (Z2-B), (Z2-S), and (Z3-B) at 1400 °C, respectively and EDAX analysis (**B4**) for sintered sample (Z2-B)

Figure 20A–D showed the SEM micro images, porosity percentage, and pore size distribution for sintered samples (Z0-B), (Z2-B), (Z2-S), and (Z3-B) at 1400 °C, respectively. The microstructure of the sample (Z0-B), which contained cordierite and mullite nanoparticles, is exhibited in Fig. 20A1 as spherical (point 1) and indefinite shapes (point 2), respectively. Also, Fig. 20B1 showed the microstructure of the sample (Z2-B) containing bagasse ash, which comprised cordierite nanoparticles as spherical shapes (point 1), mullite nanoparticles (point 2), and nano ZrO_2_ as white spots (point 3). The chemical structure of the sample (Z2-B) was also studied by EDAX analysis and showed that the main elements in that sample were (Al, Si, Mg, Zr, and O) and the composition percentages were (15.89, 10.74, 2.78, 26.69, and 43.92) %, respectively as revealed in Fig. 20B4. Similarly, sample (Z2-S) containing sawdust ash revealed the same microstructure of spherical nano cordierite (point 1), mullite (point 2), and white spots of nano ZrO_2_ (point 3) as indicated in Fig. 20C1. Figure 20D1 for the sample (Z3-B) showed only the presence of the spherical shape of nano cordierite (point 1) and white spots of nano ZrO_2_ (point 3). Therefore, the results of the sample’s microstructure supported the sample’s chemical composition and demonstrate compatibility with the XRD results. The image analysis was carried out using Digital Surf’s Mountains Lab to find out a theoretical result about the distribution of pore size and total pore area of the samples. The total pore area and histogram for the pore size range were displayed in Fig. 20(A2, A3)–(B2, B3)–(C2, C3)–(D2, D3) for sintered samples (Z0-B), (Z2-B), (Z2-S), and (Z3-B) at 1400 °C, respectively. The total area of pores was (37.13, 35.15, 39.34, and 12.78) %, as shown in Fig. 20A2, B2, C2, and D2, respectively, and the average pore size was (0.085, 0.096, 0.1019, 0.115) µm, as shown in Figure 20A3, B3, C3, D3, respectively.

### Contact angle

By using a goniometer apparatus, the water contact angles made by a sessile drop of water on a uniformly smooth surface of sintered samples at 1400 °C (Z2-B) and (Z2-S) were performed. As shown in Supplementary Fig. 1 (A), the measured contact angle of the sample (Z2-B) was 41.80°, which indicated the hydrophilic nature of that sample containing bagasse ash. While the sample (Z2-S) displayed a contact angle of 0° which proved the super hydrophilic nature ( contact angle is less than 5°) of that sample containing sawdust ash in Supplementary Fig. 1 (B) [64]. As a result of the earlier measurements, the application of sintered samples (Z2-B) and (Z2-S) at 1400 °C as ceramic membranes for water filtering was recommended [65]. This was due to the outstanding characteristics of these samples (high porosity, consistent pore size, moderate bulk density, gratified strength, and super hydrophilicity).

### Comparison of the results to previous study results

Table 3 shows the comparison between our current study with the previous studies related to cordierite–mullite-zirconia porous ceramic composites including (porosity, cold crushing strength, and bulk density).

**Table 3:** Comparison of our results with the porosity, cold crushing strength, and density of porous cordierite-mullite-zirconia with commercial products of several industries

Materials	Preparation technique	Cordierite	Mullite	ZrO_2_	Sintering temperature (°C)	BD (g/Cm^3^)	AP (%)	CCS (MPa)	Refs.
Cordierite–zirconia	Uniaxial pressure at 50 MPa	100	–	10–40	1250	2.49–2.80	28.2–2.8	–	[66]
SiC/cordierite	Pressed at 50 MPa into rectangular bars	40	–	–	1200–1400	–	59.6–27.6	9.3–54.6	[67]
Cordierite –mullite	Pressed at 50 MPa	32–51	53 − 43	–	1370–1450	–	48 − 44	9–50	[68]
Cordierite–mullite bonded porous SiC ceramics	Uniaxially pressed at 24 MPa	NM	NM	–	1400	–	40	47	[69]
Porous silica-bonded silicon carbide	Uniaxially pressed into rectangular hexahedrons at 20 MPa	–	–	–	1400	–	40	65	[70]
Silica/mullite/SiC	Uniaxially pressed at 50 MPa	–	NM	–	1400–1500	–	45.6- 36.4	10.8–39.6	[71]
Cordierite-bonded porous SiC ceramics	Pressed at 56 MPa	NM	–	–	1200–1400	–	65.69–28.07	2.9–70.3	[72]
Cordierite–zirconia	Uniaxially pressure at 100 MPa	100	–	10	1250–1400	1.77–1.85	36.51–33.36	60.69-148.18	[58]
Cordierite-mullite-zirconia	Uniaxially pressure at 79 MPa	70	30 − 0	0–30	1350–1400	1.42–1.95	55.92-46	3.26–31.35	Present work

* *NM* not mentioned

## Conclusion

- Sol-gel method was used to prepare cordierite and mullite nanoparticles, which were investigated using XRD, TEM, and BET techniques. The co-precipitation method was used to synthesize the nano t-ZrO_2_, which was then confirmed using the same techniques as before. The XRD results showed that 15 wt% of ceria is most suitable to stabilize pure t-ZrO_2_, while.
- m-ZrO_2_ was the dominant phase at less than 15 wt% of ceria.
- In general, preparing distinct ceramic phases such as cordierite and mullite via solid state reaction requires a greater firing temperature than preparing them in nanosize. Mullite phase, for example, needed a sintering temperature ranging from 1200 to 1400 °C to create it using the solid-state reaction method, whereas it was synthesized in nanosize at 1000 °C. Furthermore, nanoparticles have a very large surface area to volume ratio, which improves sintering of the ceramic composite at lower sintering temperatures. As a result, the nanoparticles will reduce the temperature of sintering, reducing energy costs.
- The samples were sintered at 1350, 1375, and 1400 °C with a constant addition of 10 wt% pore-forming agent (bagasse ash or sawdust ash) to the various cordierite, mullite, and t-ZrO_2_ ratios.
- The XRD patterns showed that cordierite, mullite, t-ZrO_2_, and m-ZrO_2_ were the dominating phases at all sintering temperatures, with the exception of a composite having 70% cordierite and 30% zirconia, which contained zircon.
- The BD of sintered composites improved with higher sintering temperatures and the addition of nano t-ZrO_2_ at the expense of nano mullite. Samples with 70 wt% cordierite and 30 wt% zirconia showed significant improvement due to the formation of the zircon phase.
- Because sawdust ash contains more carbon (97.49%) than bagasse ash (56.18%), the percentage of AP in sintered samples containing sawdust ash was higher.
- The CCS of sintered samples improved with higher sintering temperatures and nano t-ZrO_2_ additions. The samples containing bagasse ash had higher CCS values than the samples containing sawdust ash. Bagasse ash and sawdust ash sintered samples had CCS values ranging from 5.40 to 31.35 MPa and 3.26 to 7.56 MPa, respectively.
- The microstructure of selected samples (Z0-B, Z2-B, Z2-S, and Z3-B) sintered at 1400˚C showed a spherical cordierite phase, an undefined shape of mullite, and white spots of ZrO_2_. In addition, software was used to estimate the total pore area and pore size distribution (Digital Surf’s Mountains Lab and ImageJ 1.53e/Java 1.8.0_172). The pore area ranged from 12.78 to 39.34%, with an average pore size of 0.085 to 0.115 μm.
- Sample Z2 sintered at 1400 °C with bagasse ash and sawdust ash, containing 70 wt% nano cordierite, 10 wt% nano mullite, and 20 wt% nano t-ZrO_2_, showed better AP, BD, CCS, and an average pore size of 48.8%, 1.7 g/cm^3^, 16.14 MPa, and 12.77 μm, and 53.4%, 1.6 g/cm^3^, 5.06 MPa, and 12.07 μm, respectively.
- The Z2-B and Z2-S samples were found to be hydrophilic and superhydrophilic, with water contact angles of 41.80° and 0°, respectively, making them appropriate for water filter fabrication.
- The utilization of synthesized nanopowders requires a higher initial cost relative to traditional raw materials; however, the resultant ceramic composites demonstrate a distinctive amalgamation of elevated porosity, customized strength, and functional hydrophilicity, rendering them appropriate for high-value applications, including advanced filtration and catalysis. The incorporation of zero-cost waste materials as pore-formers provides notable sustainability and economic benefits to the composition.

## Supplementary Information

Below is the link to the electronic supplementary material.

Supplementary Material 1.

## Data Availability

All the authors confirm that the data supporting the findings of this study are available within the article and its supplementary materials.
