# Supplementary material for: Effect of plant waste materials as pore-forming agents on the preparation and characterization of macroporous cordierite–mullite–zirconia ceramic composites
Source: BMC Chem. 2025 Dec 19;20(1):15. doi: 10.1186/s13065-025-01696-8 (PMC12829238; doi:10.1186/s13065-025-01696-8)
Supplement: Supplementary file 1 — Supplementary Material 1. [file 13065_2025_1696_MOESM1_ESM.docx]

## Effect of plant waste materials as pore-forming agents on the preparation and characterization of macroporous cordierite- mullite- zirconia ceramic composites

## *Nadeen Nasser^1^,* *Mohamed M.S. Wahsh^3^,  Mahmoud S Rizk^1^, Gehad G. Mohamed^1,2^, Omar A. Fouad ^1^**

1. Chemistry Department, Faculty of Science, Cairo University, 12613, Giza, Egypt.
2. Nanoscience Department, Basic, and Applied Sciences Institute, Egypt-Japan University of Science and Technology, New Borg El Arab, Alexandria, 21934, Egypt
3. Refractories, Ceramics and Building Materials Department, National Research Centre, 12622, Cairo, Egypt

**Omar A. Fouad*^1*^*: The** **corresponding author**

E-mail: [oahmed@sci.cu.edu.eg](mailto:oahmed@sci.cu.edu.eg)

1. **Materials**

In this research, the used materials were aluminum chloride hexahydrate (purity 98%, Sigma-Aldrich Chemie GmbH), magnesium chloride hexahydrate (purity 99%, Sigma-Aldrich Chemie GmbH), tetraethyl orthosilicate TEOS (purity 98%, Sigma-Aldrich Chemie GmbH), zirconyl chloride octahydrate (purity 98%, Sigma-Aldrich Chemie GmbH), cerium nitrate hexahydrate (purity ≥ 98%, Fluka Guarantee), ethanol (purity 95%, Sigma-Aldrich Chemie GmbH), and ammonia solution (NH_4_OH Riedel-deHaen, Germany) were consumed to prepare nano cordierite, mullite and tetragonal zirconia. As well as bagasse ash (unprocessed sugarcane bagasse acquired from a boiler in an Egyptian sugar refinery in Minya), and sawdust ash (raw sawdust ash gained from Furniture Factory in Egypt). In detail, Table 1 displays the elemental analysis of both bagasse ash and sawdust ash. The section on instrumentation is covered in the supplementary materials.

Supplementary Table 1. The chemical composition (wt %) of pore-forming agents (bagasse ash and sawdust ash).

| oxides | Bagasse ash calcined at 700 °C | Sawdust ash calcined at 500 °C |
| --- | --- | --- |
| SiO_2_ | 27.72 | 0.27 |
| CaO | 2.50 | 0.73 |
| Al_2_O_3_ | 5.20 | ــــــ |
| TiO_2_ | ـــــــ | 0.0186 |
| P_2_O_5_ | 1.82 | 0.036 |
| SO_3_^2−^ | 0.02 | 0.047 |
| Na_2_O | 0.48 | ــــــ |
| FeO | ــــــ | 0.1297 |
| Fe_2_O_3_ | 4.40 | 0.1447 |
| K_2_O | 0.22 | 0.094 |
| Ag_2_O | ــــــ | 0.025 |
| MgO | 1.46 | ــــــ |
| MnO | ــــــ | 0.055 |
| MoO_3_ | ــــــ | 0.745 |
| Nb_2_O_5_ | ــــــ | 0.066 |
| SrO | ــــــ | 0.0354 |
| ZnO | ــــــ | 0.02 |
| ZrO_2_ | ــــــ | 0.058 |
| UO_2_ | ــــــ | 0.0185 |
| UO_3_ | ــــــ | 0.01185 |
| U_3_O_8_ | ــــــ | 0.0135 |
| Carbon | 56.18 | 97.49 |

1. **Instruments**

With Ni-filtered Cu K radiation (= 1.5406), the phase composition of the generated nanoparticles and sintered samples was determined using a Bruker D8 Discover X-ray diffractometer. In gas adsorption studies, N_2_ has been used as the adsorptive gas to determine the BET surface area at 77 K. Before the adsorption test, the materials were evacuated under a high vacuum for four to twelve hours. The calculation was based on the Brunauer-Emmett-Teller (BET) hypothesis, and the analysis was carried out using a Nova Touch LX2 analyzer. The bulk density (BD) and apparent porosity (AP) of sintered samples were calculated using the Archimedes method. Linear change (%) = [(E-E′)/E] ×100 was used to calculate the linear change of the samples before and after sintering, where E and E′ are the diameters of the samples before and after firing at high temperatures, respectively. The cold crushing strength (CCS) of every sample was determined using a hydraulic machine (SEIDNER, Riedlinger, Germany) with a maximum loading capacity of 600 kN. The pore size distribution of sintered porous ceramic samples burned at 1400 °C was measured using a mercury porosimeter (Pore Sizer, Micromeritics model 9320, USA). With a scanning electron microscope (SEM) of cracked surfaces using the Philips XL30 model, an accelerating voltage of 30 kV, magnification up to 400000, and resolution for W [3.5 nm], the microstructure and pore size distribution of a few selected samples were investigated. Before testing, samples received a thin layer of gold coating. The shape and size of the generated nanoparticles were examined using transmission electron microscopy (TEM; JEOL JEM-2100, Tokyo, Japan).


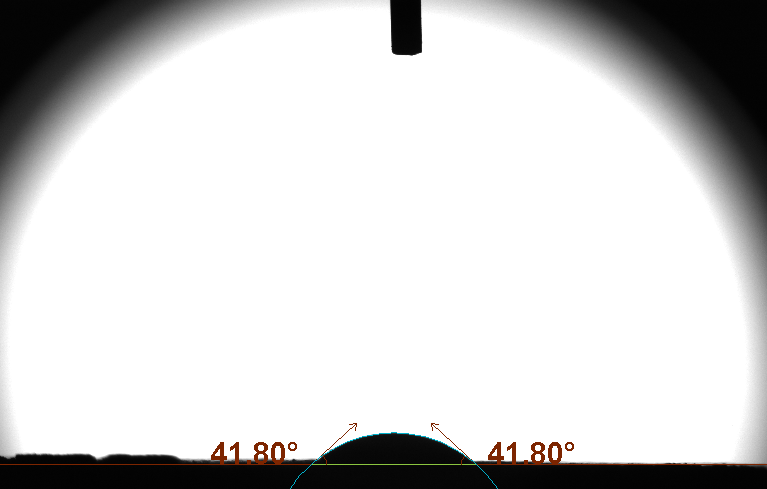

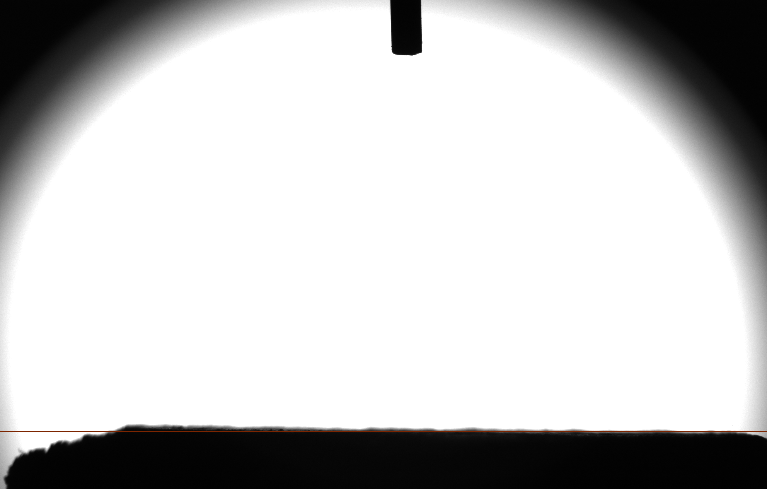


A

B

Supplementary Figure 1 (A, B). shows the contact angles of samples (Z2-B) and (Z2-S) which were sintered at 1400 ^o^C, respectively.
